# Preexisting IgG forms immune complexes and links local thermal reactogenicity with immunogenicity in influenza vaccination

**DOI:** 10.1038/s41541-026-01477-x

**Published:** 2026-05-02

**Authors:** Julia R. Hirsiger, Silke Scarascia, Mike Recher, Glenn Bantug, Christoph T. Berger

**Affiliations:** 1 Department of Biomedicine, Translational Immunology, University of Basel, Basel, Switzerland; 2 Department of Clinical Research, University Hospital Basel, Basel, Switzerland; 3 University Center for Immunology, University Hospital Basel, Basel, Switzerland; 4 Department of Biomedicine, Immunodeficiency Lab, University of Basel, Basel, Switzerland; 5 Department of Biomedicine, Immunobiology Lab, University of Basel, Basel, Switzerland

**Keywords:** Immunology, Microbiology

## Abstract

Pre-existing pathogen-specific antibodies shape vaccine outcomes, yet their impact on local reactogenicity and qualitative features of the immune response are not fully defined. In this prospective human cohort receiving seasonal influenza vaccination, high baseline hemagglutinin-specific IgG1 levels were associated with more pronounced local thermal responses at the vaccinated arm and greater vaccine-induced antibody levels. These IgG antibodies formed immune complexes with hemagglutinin, activated complement and enhanced Fc-receptor-dependent monocyte activation and phagocytosis in vitro, connecting pre-existing immunity to innate activation and local reactogenicity. Despite higher antibody levels and early plasmablast responses in subjects with strong thermal reactogenicity after vaccination, we observed lower avidity and hemagglutinin-inhibition capacity, suggesting extrafollicular responses. T cell responses were unaltered. These findings support a model in which pre-existing hemagglutinin-specific IgG may contribute to local thermal reactogenicity and qualitative features of the vaccine response through immune complex-mediated pathways, providing a framework for how prior immunity may shape human vaccine responsiveness.

## Introduction

Vaccination induces a pathogen-specific immune response to prevent subsequent infections. Protection is conferred by humoral B-cell and T-cell responses. Vaccine-specific B cells emerge from both follicular and extrafollicular pathways, leading to the development of either long-lived plasma cells and memory B cells, or short-lived antibody-secreting cells that provide rapid, yet temporary, immunological protection^1^. The determination of B cell response fate, whether follicular or extrafollicular, depends on the antigen type, T cell help, cytokine environment, and pre-existing memory from prior antigen exposure^1,2^. Generally, primary immune responses are characterized by extrafollicular plasmablasts that produce low-affinity antibodies, while some B cells migrate to germinal centers to undergo affinity maturation. Secondary responses involve follicular memory B cells that rapidly differentiate into plasma cells producing higher-affinity antibodies^3,4^. Humans are frequently exposed to respiratory viruses. For influenza, it is estimated that most individuals become infected at least once over six seasons^5^. Therefore, the seasonal influenza vaccine encounters a primed immune system with pre-existing antigen-specific antibodies and memory B cells^6^. Importantly, humoral responses to influenza vaccination can be diminished or enhanced by pre-existing immunity, depending on the antigen and the functional characteristics of the pre-existing humoral immunity^6^.

While antibody-mediated protective immunity is primarily provided by neutralizing antibodies that block the virus from infecting cells^1^, it became evident that non-neutralizing antibodies exert various protective or modulatory functions in the vaccine response. Through Fc-dependent pathways, such as antibody-dependent cellular phagocytosis (ADCP), cellular cytotoxicity (ADCC) or through complement activation (ADCA), these antibodies can label virions for uptake, shape antigen processing, and influence downstream adaptive responses^7,8^. There is increasing evidence that non-neutralizing anti-hemagglutinin (HA) antibodies contribute to influenza vaccine-induced protection^9–11^. In natural infections and immunizations with replication-competent viruses (e.g., vector or live attenuated vaccines), pre-existing immunity accelerates viral or vaccine virus clearance and can, in rare cases, facilitate antibody-dependent disease enhancement^12,13^. In contrast, vaccinations containing viral protein antigens rely on a high amount of antigen to surpass the antigenic threshold for effective immune priming^14^. FcR-dependent interactions of serum IgG with a protein antigen have been described, including the formation of immune complexes^15,16^. However, the extent to which distinct pre-existing anti-influenza antibody profiles can influence vaccine antigen uptake via Fc-mediated functions and, subsequently, the induction of the adaptive immune response to influenza vaccination, is poorly understood. Furthermore, how antibody-Fc-receptor-mediated pathways of innate immune activation contribute to reactogenicity has not been thoroughly examined.

Local injection site reactions (ISR) are the most common adverse events following immunization. The local inflammation associated with ISR is mediated by innate immune activation, a crucial element in initiating the immune response to vaccines. Vaccine-mediated activation of pathogen- or damage-associated molecular patterns (PAMPs and DAMPs) leads to the release of pro-inflammatory cytokines, cell migration, and antigen uptake via endocytosis or phagocytosis^17^. Local inflammation arises a few hours after vaccination and usually resolves within three days^17^. Pre-existing vaccine-specific immunity contributes to the vaccine reactogenicity, as evidenced by severe local reactions (“hyperimmunization”)^18^ or immune-complex-mediated reactions (Arthus reactions or type III hypersensitivity reactions)^19^. Few studies have investigated whether the reactogenicity of the influenza vaccine is associated with vaccine immunogenicity or efficacy. In heart failure patients, adverse events following influenza vaccination were associated with better cardiovascular outcomes^20^. A post-hoc analysis of an immunogenicity study observed stronger local reactions in subjects with low pre-existing immunity but a high antibody fold increase^21^. In studies of COVID-19 vaccines, fever, but not local reactogenicity, was associated with higher antibody levels^22–25^. Notably, none of these studies monitored local temperature reactions.

Here, we studied the contributing immunological factors and consequences of local temperature reactions to seasonal influenza vaccination in a human model system with variable pre-existing immunity and reactogenicity.

## Results

### Influenza vaccination induced a local thermal reaction 24 h post-vaccination

**Fig. 1:**
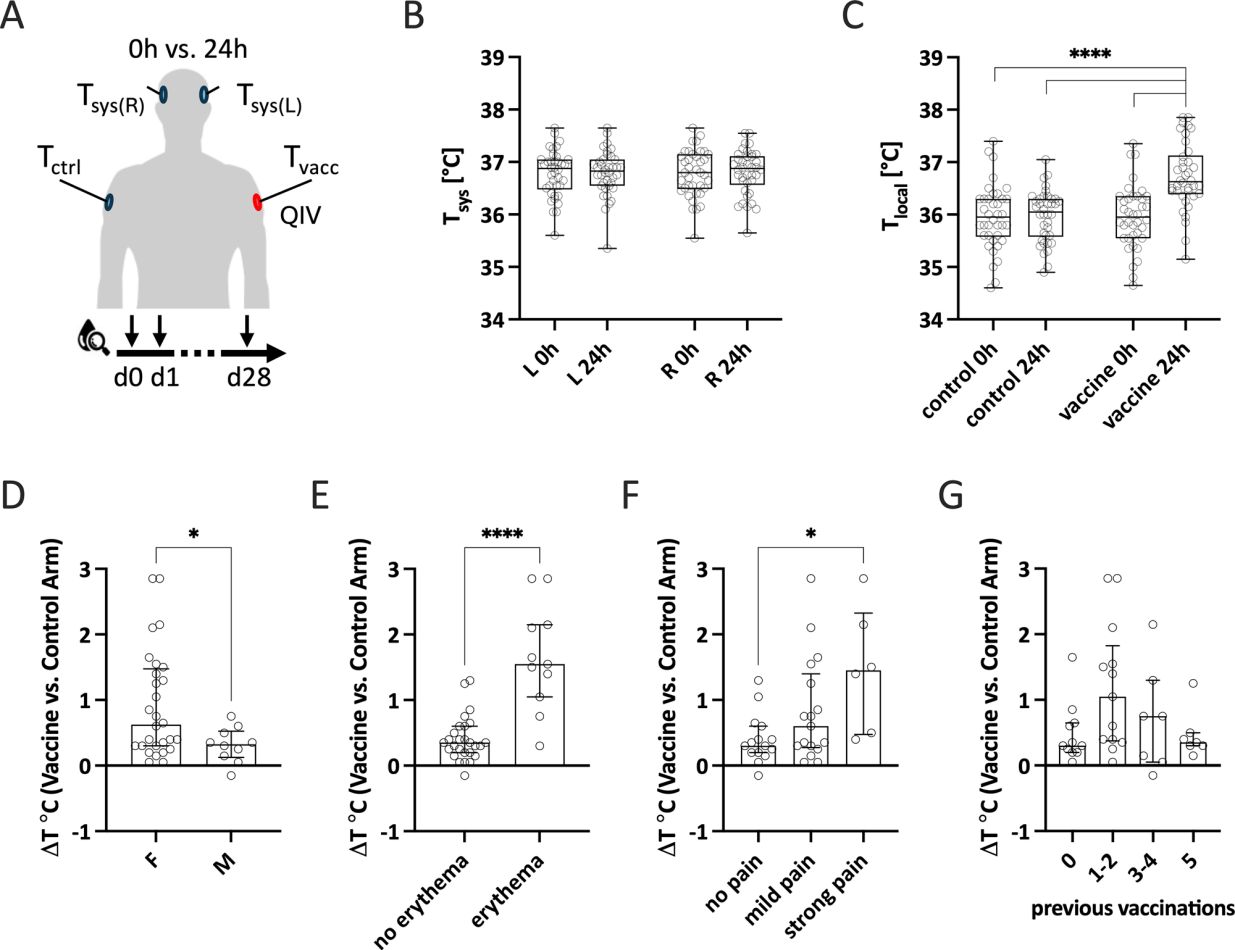
Clinical correlates of the local thermal reactogenicity. **A** Schematic of systemic (temporal) and local (deltoid) temperature measurements. **B** Systemic temperature (in °C) measured before (0 h) and 24 h post-vaccination (24 h) at the left (L) and right (R) temporal regions. **C** Local temperatures at the unvaccinated (control) and vaccinated (vaccine) arms are indicated at the pre- (*n* = 34) and 24 h post-vaccination (*n* = 38) time points. Box plots display the median and 25th–75th percentiles and whiskers the 10th–90th percentiles. Local temperature reactions (median and interquartile range) stratified by **D** sex, **E** presence of erythema, **F** pain, and **G** the number of prior vaccinations in the last 5 years. Statistical comparisons were performed using Wilcoxon signed-rank or Mann-Whitney *U* tests as appropriate. *p < 0.05, ****p < 0.0001. QIV quadrivalent influenza vaccine; °C degrees Celsius, T_sys_ systemic temperature, T_vacc_ temperature at the vaccinated arm, T_ctrl_ temperature at the control arm, M men, F women.

Between November 2019 and January 2020, 38 healthy subjects who received the inactivated seasonal influenza vaccination were enrolled. The baseline characteristics of the study population are summarized in Table 1. We measured temperatures at the temporal region (“systemic temperature”) and the deltoid region (“injection site” and “control site”) before and 24 h following quadrivalent inactivated influenza vaccination (QIV) using a no-touch temporal thermometer (Fig. 1A). The increase in skin surface temperature at the injection site was defined as the “local thermal reaction/reactogenicity”. Systemic temperature was unchanged 24 h post-vaccination and comparable between the two temporal measurement sites (baseline median *T*_temp(L)_ 36.85 °C vs. *T*_temp(R)_ 36.80 °C, *p* = 0.90; 24 h post-vaccination median *T*_temp(L)_ 36.83 °C vs. *T*_temp(R)_ 36.88 °C, *p* = 0.98) (Fig. 1B). Vaccination induced a significant temperature increase at the vaccinated arm 24 h post-vaccination (median *T*_vacc_ 36.63 °C vs. 35.90 °C; *p* < 0.0001) but not at the control arm (median *T*_ctrl_ 36.05 °C vs. 35.95 °C; *p* = 0.73) (Fig. 1C). The median temperature increase at the vaccinated arm was higher in women (ΔT_vacc24h-BL_ + 0.63 °C vs. +0.32 °C in men, *p* = 0.04) (Fig. 1D) and in those with a clinically observed local erythema (ΔT_vacc24h-BL_ + 1.55 vs. +0.35 °C, *p* < 0.0001) (Fig. 1E). The erythema diameter was correlated with the ΔT at the vaccine arm (Supplementary Fig. 3, *p* < 0.0001, *r* = 0.68), and self-reported severe pain showed a significant correlation with the temperature increase (Fig. 1F). The number of influenza vaccines received in the preceding five years had no influence (Fig. 1G).

**Table 1:** Baseline characteristics

Total	*n* = 38
Women, *n* (%)	27 (70.5%)
Age, median (IQR)	31 (27-42.5)
BMI, median (range)	21.9 (20.7-24.1)
Vaccinated arm	
left	34 (89.5%)
right	4 (10.5%)
Medical history	
Allergy^a^	15 (39.5%)
Autoimmunity^b^	3 (7.9%)
Current Smoker	1 (2.6%)
ILI past 5 years	
none	27 (70.5%)
previous year only	1 (2.6%)
>1–5 years ago	10 (26.3%)
Influenza vaccines (past 5 years)	
0	11 (28.9%)
1	11 (28.9%)
2	2 (5.3%)
3	4 (10.5%)
4	3 (7.9%)
5	7 (18.4%)

*ILI* influenza like illness.

^a^RCA allergic rhinoconjunctivitis, asthma, penicillin.

^b^Basedow (*n* = 1), Psoriasis (*n* = 2).

### Association between distinct pre-vaccination immune profiles and a subsequent strong local temperature reaction

**Fig. 2:**
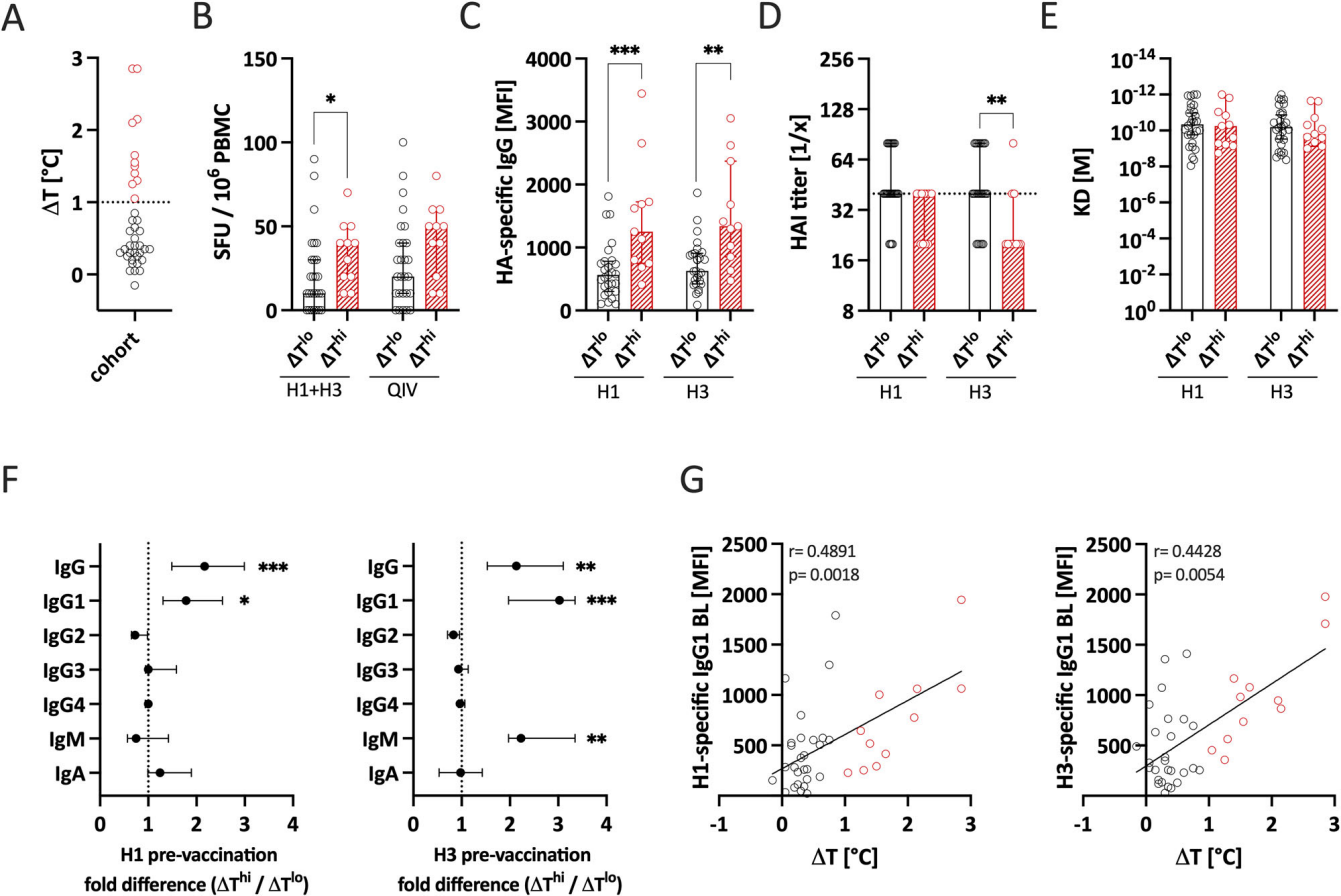
Association of pre-vaccination immune profiles with thermal local reactions. **A** A visual demarcation of the ΔT threshold (*n* = 38) at 1 °C (dashed line) to divide the cohort into individuals with elevated temperature responses (ΔT^hi^) compared to those with a mild thermal reaction (ΔT^lo^). **B** Influenza-specific T cell response measured by IFNγ-Elispot (SFU/M = spot-forming units per million PBMC). **C** Pre-vaccination HA-strain-specific IgG in Luminex and **D** neutralization titer in HA-inhibition assays. **E** Binding affinity measured by biolayer interferometry (BLI) against H1 and H3 expressed as equilibrium dissociation constant (KD) in Molar (M). Data are presented as median ± IQR (**A**–**E**) with *n* = 27 for ΔT^lo^ and *n* = 11 for ΔT^hi^ in all experiments. **F** IgG, IgA, IgM, and IgG_1-4_ subclass contributions to the HA-binding antibodies are expressed as the median ratio ΔT^hi^/ΔT^lo^ ± upper/lower limits (i.e., values > 1 indicate higher antibody levels of this subclass in ΔT^hi^). *n* = 27 for ΔT^lo^ and *n* = 11 for ΔT^hi^. **G** Correlation of HA-specific IgG1 levels with ΔT in absolute values (*n* = 38) by simple linear regression analysis. The *p* and *r* values were obtained using Pearson correlation. °C degrees Celsius, H1 hemagglutinin from H1N1 vaccine strain, H3 hemagglutinin from H3N2 vaccine strain, QIV quadrivalent influenza vaccine, MFI median fluorescence intensity, BL baseline. Statistical comparisons were performed using Mann-Whitney *U* tests. *p < 0.05, **p < 0.01, ***p < 0.001.

The thermal reaction at the vaccination site indicates local inflammation and may be influenced by innate immunity and pre-existing influenza-specific immunity. We explored these interactions by dividing the cohort into individuals with disproportionately elevated temperature responses compared to those with a mild thermal reaction (i.e., ΔT^hi^ vs. ΔT^lo^). We used an arbitrary threshold of ΔT ≥ +1 °C for the grouping, which aligned with a clear inflection in the empirical distribution, consistent with a shift toward a distinct physiological response pattern (Fig. 2A). First, we investigated the composition of peripheral blood immune cells. At pre-vaccination baseline, the distribution and activation status of inflammatory and non-inflammatory monocytes, as well as the frequencies of the tested CD4 T cell, CD8 T cell and B cell subsets, were comparable between the ΔT^hi^ and ΔT^lo^ group (Supplementary Fig. 4). Pre-vaccination frequencies of IFNγ-producing HA- and influenza vaccine-specific T-cells were generally low, but slightly higher in the ΔT^hi^ group (for recombinant H1/H3 HA antigen 10 vs. 40 SFC/M, *p* = 0.035; for QIV 20 vs. 50 SFC/M, *p* = 0.07) (Fig. 2B). By comparison, ΔT^hi^ subjects had higher total HA-specific IgG directed against the H1 and H3 HA of the vaccine strains (*p* = 0.001 for H1 and *p* = 0.0013 for H3) (Fig. 2C). In contrast, pre-vaccination sera from ΔT^lo^ subjects exhibited a more potent hemagglutination inhibition capacity against the H3 vaccine strain (Fig. 2D). Antibody avidity, assessed as the dissociation rate by biolayer interferometry^26^, was comparable between groups (Fig. 2E). HA-specific antibodies belonging to the IgG1 subclass accounted for the observed difference in the anti-HA-IgG levels between the two groups (anti-HA-IgG1 1.79-fold higher in the ΔT^hi^ for H1 (*p* < 0.027) and 3.02-fold higher for H3 (*p* < 0.001)) (Fig. 2F). The absolute levels of HA-specific IgG subclasses and classes are presented in Supplementary Fig. 5, showing that only HA-specific IgG1 levels were significantly higher. H3-specific IgM was relatively higher in ΔT^hi^ than in ΔT^lo^, but absolute IgM values were negligibly low. HA-specific IgG1 levels were correlated with the local ΔT across the whole cohort (H1: *r* = 0.49, *p* = 0.002; H3: *r* = 0.44, *p* = 0.005) (Fig. 2G). To ensure that the observed differences between the ΔT^hi^ and ΔT^lo^ groups were independent of the chosen cut-off to segregate the groups, we performed a complementary unsupervised cluster analysis. Hierarchical clustering confirmed associations between baseline immune characteristics, demographic and clinical factors, with the local thermal reaction (Supplementary Fig. 6).

Combined, we found that pronounced thermal reactogenicity was associated with higher pre-existing anti-influenza antibody levels, and that the difference was driven explicitly by elevated pre-vaccination HA-specific IgG1 levels.

### Pre-existing IgGs from subjects with subsequently augmented thermal reactogenicity potently activated monocytes

**Fig. 3:**
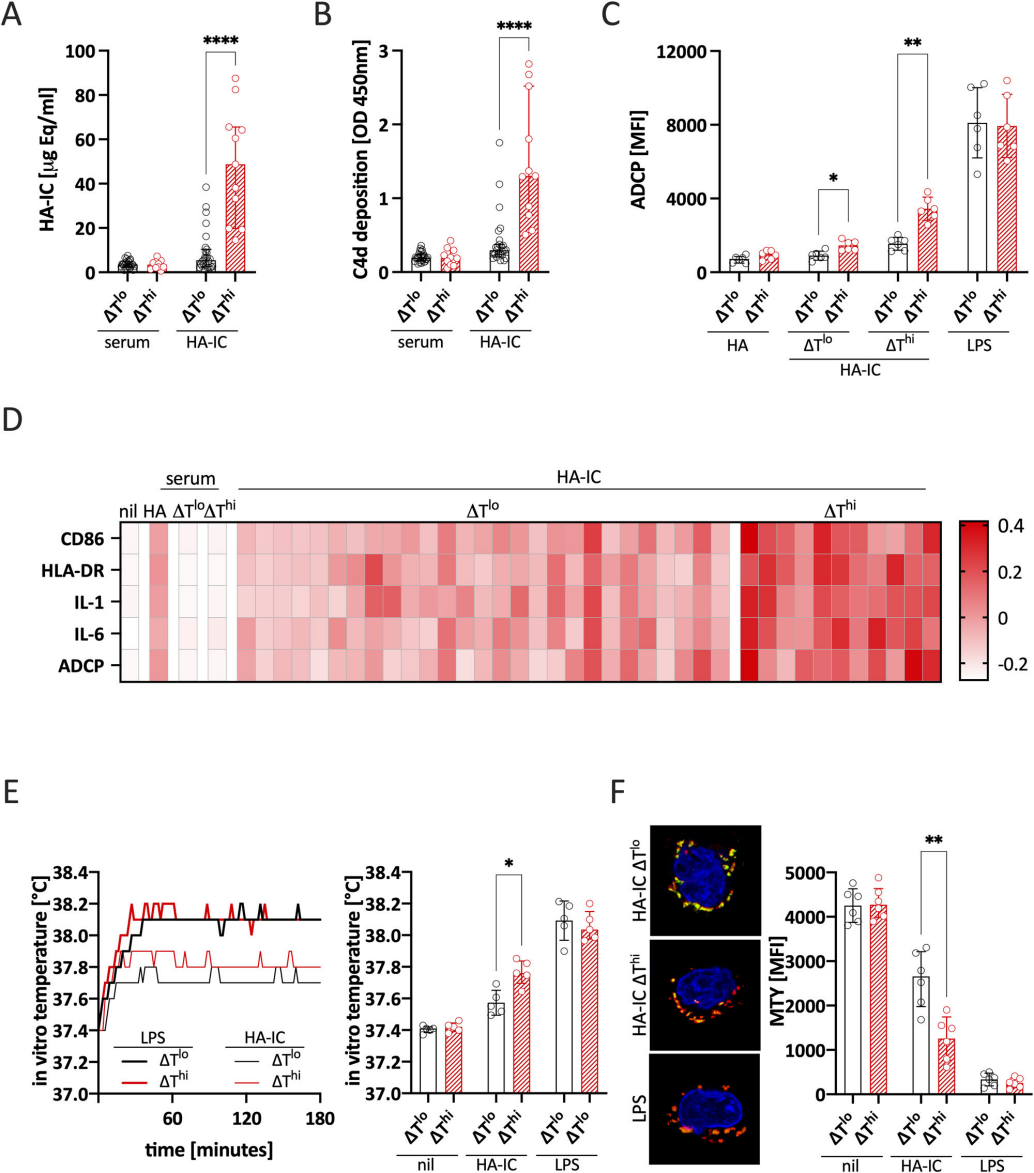
FcγR-mediated monocyte activation by HA-IgG immune complexes. **A** C1q binding of HA-IgG-IC formed by pre-vaccination sera of ΔT^hi^ (*n* = 11) and ΔT^lo^ (*N* = 27) subjects. Data are presented as median ± IQR. **B** ELISA-based measurement of C4d deposition on HA–IgG-IC formed with pre-vaccination sera from ΔT^hi^ (*n* = 11) and ΔT^lo^ subjects (*n* = 27) to assess activation of the classical complement pathway. **C** Comparison of spontaneous, ADCP-mediated, and TLR-stimulated HA phagocytosis in ΔT^hi^ vs. ΔT^lo^ monocytes. Pooled data of *n* = 3 independent experiments with two donors per group and experiment (*n* = 6 ΔT^lo^, *n* = 6 ΔT^hi^). Data are presented as median ± IQR (**A–C**). **D** Heatmap summarizing immune complex-mediated activation (CD86/HLA-DR), cytokine (IL-6/IL-1) secretion, and antibody-dependent phagocytosis (ADCP) in the THP-1 monocyte cell line incubated with sera or HA-IC from ΔT^hi^ (*n* = 11) and ΔT^lo^ subjects (*n* = 27). Color scale represents vector-scaled values, obtained by dividing each value by its Euclidean norm (√∑x²). nil unstimulated THP-1, HA hemagglutinin alone. **E** Example temperature curve (left) and summary graph (right) of cellular heat production following IC-mediated activation in THP-1 cell cultures measured using a thermal probe. Pooled data of *n* = 5 independent experiments with two donors per experiment (*n* = 5 ΔT^lo^, *n* = 5 ΔT^hi^). **F** Immunofluorescence microscopy using the thermo-sensitive MTY on ΔT^hi^ vs. ΔT^lo^ HA-IgG-IC activated THP-1 (left) and summary graph (right). Pooled data of *n* = 3 independent experiments with two donors per group and experiment (*n* = 6 ΔT^lo^, *n* = 6 ΔT^hi^). Data are presented as median ± IQR (**F**, **G**). Statistical comparisons were performed using Mann-Whitney *U* tests and one-way ANOVA with Dunn’s multiple comparison test as appropriate. *p < 0.05, **p < 0.01, ****p < 0.0001. HA-IC immunocomplexes formed from recombinant hemagglutinin and serum IgG, QIV quadrivalent influenza vaccine, LPS lipopolysaccharide (TLR4 agonist), °C degrees Celsius, MTY MitoThermo Yellow.

Local injection-site reactions result from vaccine- or adjuvant-mediated innate immune activation. Immune complexes can trigger cytokine-independent monocyte activation via the Fc-receptor (FcR) or complement receptors. IgG1, the HA-specific IgG subclass that was significantly elevated in pre-immunization sera of ΔT^hi^ subjects, potently activates FcR^27^. Accordingly, we examined whether the higher levels of anti-HA-IgG1 in ΔT^hi^ subjects were associated with enhanced FcR-mediated monocyte activation or complement system activation. To test this, recombinant hemagglutinin was mixed with pre-vaccination sera to form HA-IgG immune complexes (HA-IgG-IC). Using a C1q-binding assay, we demonstrated that HA-IgG-IC from ΔT^hi^ more robustly bound complement (Fig. 3A), which was directly correlated with the amount of HA-specific IgG (H1: *r* = 0.73, *p* < 0.0001; H3: *r* = 0.72, *p* < 0.0001). Next, we used an in vitro complement activation assay that measures CD4d deposition by ELISA^28^. HA-IgG-IC from ΔT^hi^ showed significantly stronger serum complement activation than HA-IgG-IC from ΔT^lo^ (Fig. 3B). The ADCP capacity of HA-IgG-IC formed with the pre-vaccination sera was assessed using fluorochrome-labeled hemagglutinin (HA_fluo_) to monitor cellular uptake of HA_fluo_-IgG-IC. Using primary monocytes and pre-vaccination sera from ΔT^hi^ and ΔT^lo^ subjects, we observed an increased ADCP of HA_fluo_-IgG-IC by monocytes from ΔT^hi^ subjects (Fig. 3C). In contrast, there was no difference in HA uptake after FγR-independent monocyte activation by TLR4-stimulation with LPS, nor when the same experiments were conducted with mDCs (Fig. 3C and Supplementary Fig. 7). We confirmed that IC uptake in this assay depended on a functional IgG/FcγR interaction using blocking antibodies against FcγRI (CD64), FcγRII (CD32), or FcγRIII (CD16), alone or in combination (Supplementary Fig. 7). Overall, there was redundancy among the different FcγR, but blocking FcγRI most potently decreased IC phagocytosis. Complementary experiments using IgG-depleted sera or the serum of a subject with severe hypogammaglobulinemia further confirmed that HA uptake was mainly ADCP-mediated (Supplementary Fig. 7).

Next, we aimed to compare the ADCP function of the pre-vaccination sera in a controlled cellular system using the THP-1 cell line. Stimulation of the THP-1 monocyte cell line with pre-vaccination HA-IgG-IC from ΔT^hi^ subjects induced significantly enhanced THP-1 activation (HLA-DR^hi^ CD86^hi^), pro-inflammatory cytokine secretion (IL-1β and IL-6), and antibody-dependent phagocytosis (ADCP) compared to HA-IgG-IC from ΔT^lo^ subjects (Fig. 3D). The associations between the local thermal reaction and monocyte activation were comparable regardless of whether H3 or H1 was used as the hemagglutinin target (Fig. 3D and Supplementary Fig. 8) and were confirmed using the absolute ΔT values in complementary correlation analyses (Supplementary Fig. 8). ADCP and monocyte activation were closely connected to the levels of pre-existing HA-specific IgG1, the amount of HA-IgG-IC formed in vitro, and the clinically observed local thermal reactogenicity, which showed the strongest statistical correlation (Supplementary Figs. 9 and 10).

In vivo, we observed differences in the frequency of peripheral blood monocytes amongst PBMC 24 h post-vaccination in ΔT^hi^ vs^.^ ΔT^lo^ (median 10.0% vs. 17.7%, *p* = 0.009), with lower frequencies of classical (CD14^high^CD16^neg^) monocytes (44.7% vs. 62.8%, *p* = 0.004) and an increased frequency of non-classical (CD14^low^CD16^high^) monocytes (29.4% vs. 10.5%, *p* = 0.041) in ΔT^hi^ (Supplementary Fig. 11). These monocytes exhibited a higher median fluorescence intensity (MFI) of HLA-DR expression across all monocyte subsets (CD14^high^CD16^negative^, CD14^high^CD16^low^, and CD14^low^CD16^high^), while CD86 was only elevated on CD14^high^CD16^negative^ monocytes (Supplementary Fig. 11).

Inflammation-related heat likely results from hyperemia driven by local cytokine release, but it may also stem from cellular heat generation. We investigated this by measuring the temperature of the cell culture media in stimulated THP-1 cells, cultured at 37 °C, using an immersion temperature probe. Monocytes of ΔT^hi^ subjects produced significantly more heat in vitro (T increase to 37.55 °C vs. 37.76 °C, *p* = 0.0159) when activated by autologous HA-IgG-IC, but not when non-specifically activated with LPS (Fig. 3E, F). In addition, we employed a thermo-sensitive mitochondrial-targeted fluorescent dye, MitoThermo Yellow (MTY), to visualize mitochondrial temperature production at the cellular level in primary monocytes. In this assay, higher temperature production results in reduced MTY fluorescence intensity^29^. Differences in the mitochondrial temperature production were confirmed in the MTY assay comparing ΔT^hi^ and ΔT^lo^ HA-IgG-IC-mediated THP-1 cell activation (MTY MFI 1058 vs. 2562, *p* = 0.002) (Fig. 3G). Immune cell heat generation was almost exclusively monocyte-derived (Supplementary Fig. 11).

Together, these results suggest that anti-HA IgG1 contributes to FcR -dependent and/or complement-mediated monocyte activation through ICs in vitro. This is consistent with the possibility that IC-activated innate cells contribute to local thermal reactogenicity, although this was not directly demonstrated in vivo.

### Impact of temperature on B-cell functions in vitro

**Fig. 4:**
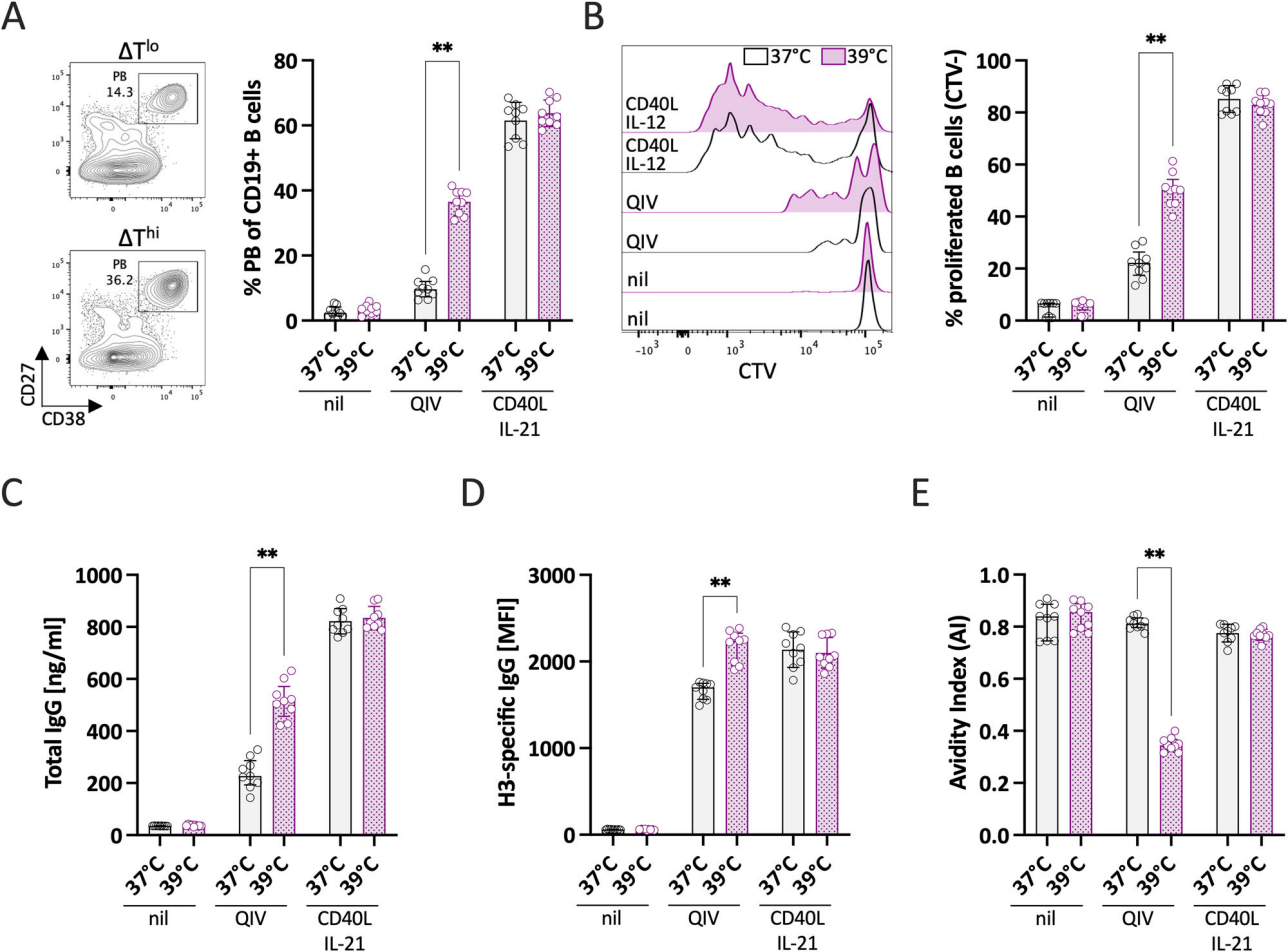
Effect of temperature on B cell function. **A** In vitro plasmablast differentiation (example FACS plot and summary graph) and **B** B cell proliferation (example CTV dilution histogram and summary graph) after PBMC stimulation with QIV at a cell culture temperature of 37 °C (gray) vs. 39 °C (purple). Frequency of PB (CD3^−^CD19^+^CD20^−^CD27^+^CD38^+^) and proliferated B cells (CTV^low^) after 5 days is shown. **C** Total IgG production (ELISA) and **D** A/Kansas/14/2017 (H3N2)-specific IgG production (Luminex) were compared at 37 °C vs 39 °C. **E** The avidity of A/Kansas/14/2017 (H3N2)-specific IgG produced at 37 °C vs. 39 °C was compared in urea challenge assays. Data represent three independent experiments, each with three different donors (*n* = 9). Data are presented as median ± IQR. Statistical comparisons were performed using Mann-Whitney *U* tests **p < 0.01. PB plasmablasts, QIV quadrivalent influenza vaccine, CTV CellTrace™ Violet, °C degrees Celsius.

Thermal energy drives several immunological processes, including cell migration, cellular activation, cytokine secretion, and cell proliferation^30–33^. Although our data establish FcR-dependent monocyte heat generation in vitro, the extent to which such micro-scale thermal changes occur in vivo and reach the B cell niches remains to be defined. Nevertheless, these findings prompted us to examine how modest, transient temperature elevations might affect antigen-driven B cell activation. We performed experiments in temperature-controlled cultures to model the impact of in vivo inflammation-associated heat, hypothesizing that immune-complex-activated innate immune cells may generate heat, as suggested by our in vitro data. To begin to explore this, we stimulated pre-vaccination PBMC with the QIV at 37 °C and 39 °C for 5 days and monitored in vitro B cell proliferation and differentiation into CD27^high^CD38^high^ plasmablasts (PB). The detailed B cell gating strategy is shown in Supplementary Fig. 1. QIV-stimulated B cells cultured at 39 °C exhibited four-fold higher PB frequencies (36.5% vs. 9.6%, *p* = 0.0039; Fig. 4A) and more than two-fold increased proliferation (50.2% vs. 20.5%, *p* = 0.0039; Fig. 4B) compared to cells cultured at 37 °C. Notably, we did not observe any effect of culture temperature when using non-BCR-mediated B cell stimulation with CD40L/IL-21 (Fig. 4A, B). Activating B cells with QIV at elevated temperatures enhanced polyclonal IgG production (Fig. 4C), resulted in a mild increase in IgM (Supplementary Fig. 12), and was associated with a significant increase in influenza strain-specific IgG (Fig. 4D). However, HA-specific antibodies produced at 39 °C had a lower avidity index in urea challenge assays than those produced at 37 °C (0.81 vs 0.34, *p* = 0.0039, Fig. 4E).

These findings suggest that an elevated temperature in the microenvironment of B cells promotes antigen-induced B cell activation, proliferation, and antibody secretion.

### A strong thermal reactogenicity associated with a high magnitude but low-avidity and poorly hemagglutinin inhibiting IgG response in vivo

**Fig. 5:**
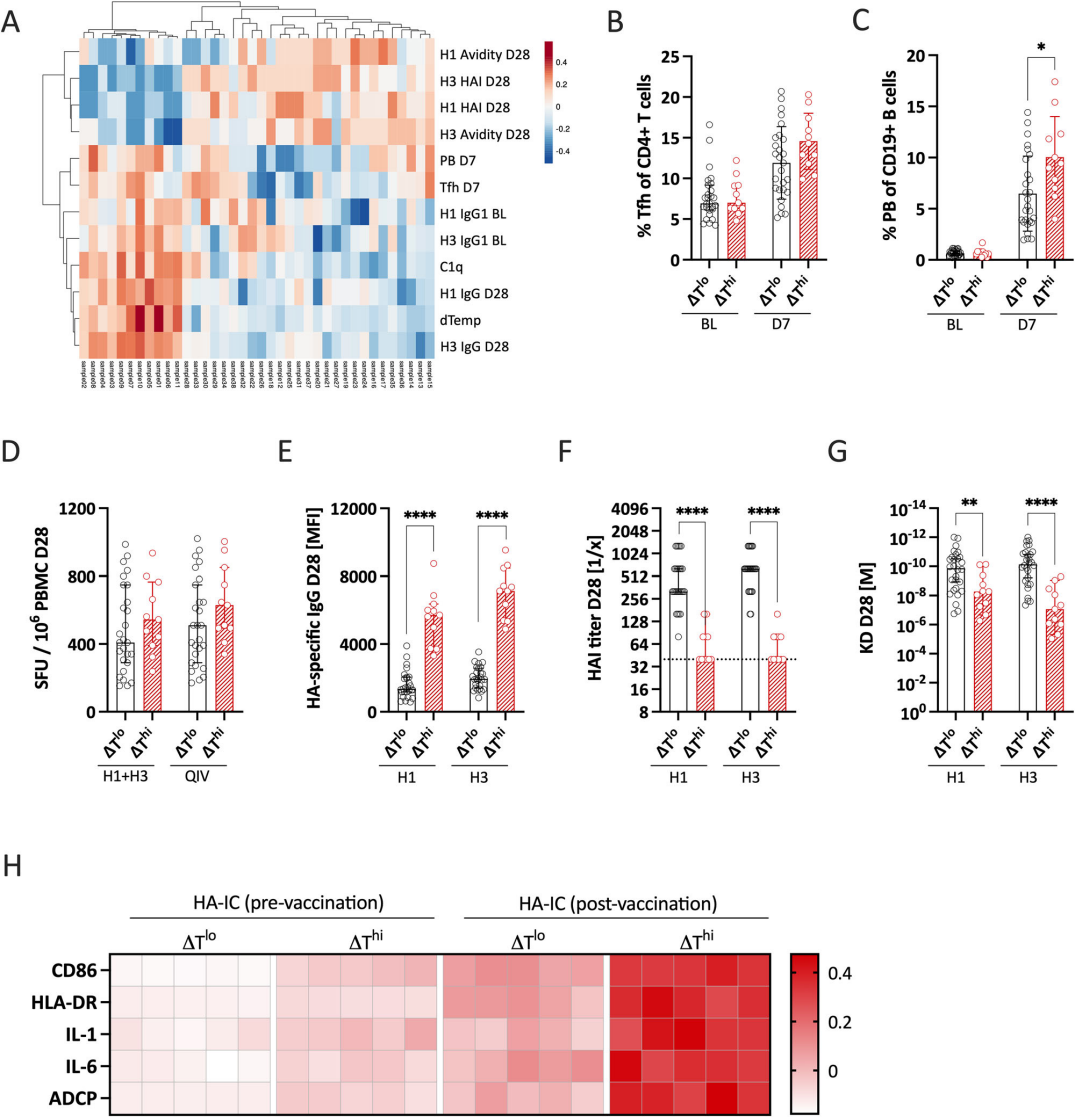
In vivo associations of local temperature reactogenicity and the adaptive vaccine response. **A** Unsupervised hierarchical clustering analysis of baseline immune profiles and the vaccine response outcomes. **B** Peripheral blood frequencies of Tfh and **C** PB seven days post-vaccination. **D** QIV- and HA-specific T cell responses at day 28 in IFNγ EliSpot expressed as SFC/M. **E** HA-specific IgG levels post-vaccination in strain-specific Luminex. **F** Neutralization titers in HA inhibition assays against the vaccine strains A/Brisbane/02/2018 (H1N1) and A/Kansas/14/2017 (H3N2). The dashed line indicates the seroprotective titer 1:40. **G** Antibody avidity in biolayer interferometry expressed as KD. Data are presented as median ± IQR in (**A**–**F**) (*n* = 11 ΔT^hi^, *n* = 27 ΔT^lo^). **H** Heatmap of non-neutralizing, Fc-mediated antibody functions tested using HA-IgG-IC formed from serum day 28 post-vaccination. Color scale represents vector-scaled values (*n* = 5 ΔT^hi^, *n* = 5 ΔT^lo^), obtained by dividing each value by their Euclidean norm (√∑x²). Statistical comparisons were performed using Mann-Whitney U tests. *p < 0.05, **p < 0.01, ****p < 0.0001. Tfh circulating follicular T helper-like T cells, PB plasmablasts, SFC/M spot-forming cells per million PBMC, HA-IC recombinant hemagglutinin/serum IgG-immunocomplexes.

The thermal reaction at the vaccination site transiently exposes immune cells to elevated temperatures. Given the in vitro evidence that heat modulates B cell activity, we next examined whether vaccine-induced immune responses differed between individuals with high (ΔT^hi^) and low (ΔT^lo^) thermal reactivity. We assessed the frequencies of plasmablasts and T follicular helper (Tfh)-like T cells in the peripheral blood on day 7, along with T cell responses, antibody levels, HAI titers, and antibody avidity on day 28. An unsupervised hierarchical clustering analysis revealed that thermal reactogenicity, pre-vaccination HA-specific IgG1, and immune complex formation were strongly interconnected and associated with features of an extrafollicular B cell response (Fig. 5A).

Subsequently, we performed direct group comparisons between the ΔT^hi^ and ΔT^lo^ groups. Plasmablast egress from lymph nodes and the expansion of circulating Tfh-like T cells are hallmarks of the early influenza vaccine response in humans^34,35^. At baseline and on day 7, Tfh-like cell frequencies were comparable across groups (Fig. 5B). In contrast, ΔT^hi^ subjects exhibited significantly higher frequencies of circulating plasmablasts at 7 days post-vaccination (Fig. 5C) and a more significant fold change compared to baseline (median: ΔT^lo^ 9.250 vs. ΔT^hi^ 15.96 fold change, p = 0.013). T cell intrinsic HA-specific IFNγ production at day 28 post-vaccination was non-significantly elevated in ΔT^hi^ subjects (H1 + H3 408 vs 544 SFC/M, *p* = 0.3; QIV 510 vs. 629 SFC/M, *p* = 0.2) (Fig. 5D). On average, the total HA-specific IgG, specifically IgG1, against the vaccine strains at 28 days post-vaccination was 4-fold higher in subjects that exhibited strong thermal reactions (range 3.67–4.22-fold higher in ΔT^hi^ subjects) and correlated with the absolute temperature increase at the vaccination site (Fig. 5E and Supplementary Fig. 13). In contrast, post-vaccination sera from subjects with mild thermal reactogenicity demonstrated greater HAI titers (as a surrogate for virus neutralization capacity) against the H1 and H3 vaccine strains (Fig. 5F). Divergence in antigen-binding and neutralization capacity may be due to differentially targeted epitopes or to differences in antibody avidity. We found that vaccination-induced influenza-specific IgGs in ΔT^hi^ subjects were of lower avidity than in those with mild thermal reactogenicity, as assessed by biolayer interferometry and urea challenge (Fig. 5G and Supplementary Fig. 13). The QIV induced HA-specific IgG of all subclasses, but subclass distributions varied by HA specificity and thermal response profile, with IgG1 dominance in ΔT^hi^ sera (Supplementary Fig. 13). We then tested the d28 post-immunization serum for HA-IgG-IC-mediated THP-1 activation. HA-IgG-IC formed with post-vaccination (day 28) sera robustly activated THP-1 and enhanced ADCP, particularly when derived from ΔT^hi^ individuals (Fig. 5H). All key findings were confirmed in complementary analyses using ΔT and the vaccine response readouts as continuous variables (Supplementary Fig. 14).

These data collectively suggest an association between increased thermal reactogenicity and higher levels of pre-existing, non-neutralizing (i.e., low HAI titers) anti-HA antibodies, which in our in vitro assays promoted the formation of IgG1-enriched immune complexes. Stronger Fcγ receptor- and/or complement-mediated activation in vitro was associated with higher antibody magnitude post-vaccination, but these antibodies had lower avidity and exerted lower hemagglutinin inhibition. Taken together, these suggest a potential model where pre-existing antibodies, IC-mediated innate activation, and local inflammation are linked to qualitative differences in the vaccine response.

## Discussion

Local reactogenicity is the most common adverse event following immunization, yet its immunological basis and consequences remain incompletely defined. In healthy adults receiving the seasonal influenza vaccine, we quantified the local temperature increase at the injection site and leveraged its broad inter-individual variability to dissect potential mechanisms underlying this response and its impact on adaptive immunity. Specifically, influenza vaccination triggered a measurable thermal reaction at 24 h, enabling classification into high (ΔT^hi^) and low (ΔT^lo^) responders.

We found that immune complexes formed between HA-specific IgG and recombinant hemagglutinin bound and activated complement in vitro. Similarly, HA-IgG-IC activated innate cells by engaging FcγR, thereby prompting monocyte activation, upregulating activation markers (HLA-DR and CD86), secreting inflammatory cytokines, and enhancing ADCP. The ΔThi subjects showed significantly higher levels of HA-specific IgG1 before vaccination. Additionally, the immune complexes with their serum exhibited significantly stronger FcγR-dependent immune activation and phagocytosis in monocytes. This underscores the role of pre-existing non-neutralizing IgG in promoting local inflammatory responses. However, while our experiments support biologic plausibility, they do not prove that the interaction of IC with complement or FcR is the dominant in vivo driver at the injection site. Our results diverge from a recent report that associated low baseline immunity with increased reactogenicity, although the lack of temperature measurements in that study limits direct comparison^21^. Moreover, we cannot exclude that other pathogen-specific IgG subclasses are more relevant than IgG1 in other vaccines or other clinical contexts.

Conceptually, the pre-existing IgG against HA, forming HA-IgG-IC following immunization with HA and causing immune activation, is consistent with a type III hypersensitivity-like (Arthus-like) reaction^19^. Unlike classical type III hypersensitivity, observed after exposure to an intravenous antigen that allows ICs to distribute to joints or other organs, the vaccine reaction is limited locally. Notably, the local thermal reaction, the baseline HA-specific IgG1, the HA-IgG-IC, and FcR-mediated monocyte activation and ADCP were closely interlinked, and the amount of HA-IgG-IC correlated with the same baseline and outcome parameters as the ΔT (Figs. S9 and 5A). Therefore, our study design prevents us from isolating the effects of immune complex-mediated complement activation and FcR-mediated monocyte activation on local heat production and reactogenicity. However, our data suggest that the overall innate activation is reflected clinically in the local thermal response, which serves as an easy, measurable, quantitative surrogate.

The second key finding was that the local thermal reactivity was associated with downstream adaptive responses. In exploratory in vitro experiments, elevated culture temperatures were associated with enhanced B cell proliferation, plasmablast differentiation, and antibody secretion, in line with emerging evidence that heat affects immune cell dynamics and trafficking^30^. However, these experiments only approximate the in vivo setting and do not establish that local temperature elevations at the injection site directly drive the observed adaptive response pattern. In vivo, ΔT^hi^ individuals mounted higher HA-specific total IgG and IgG1 responses by day 28; however, these antibodies exhibited reduced avidity in biolayer interferometry and urea-challenge assays and exhibited weaker hemagglutinin inhibition capacity, used here as a surrogate for neutralizing capacity. Collectively, these findings indicate that pronounced local thermal reactogenicity correlates with a higher-magnitude but qualitatively distinct humoral response. The mechanisms underlying this relationship still need further investigation. Non-mutually exclusive mechanisms are plausible, including effects of pre-existing antibodies on antigen availability, immune complex formation, antigen uptake, and processing.

Previous studies have reported higher local reactogenicity and immunogenicity in women; however, most examined mRNA or adjuvanted vaccines and did not quantify local thermal responses, limiting direct comparison^36^. Fever after influenza vaccination has been associated with higher hemagglutination inhibition titers^37^, however, no participants in our study developed fever. Gromer et al. reported that local reactions were associated with lower pre-existing IgG levels, although local thermal responses were not assessed^21^.

The inactivated influenza vaccine has an excellent safety profile, but local reactogenicity is common (30–50% of vaccinated subjects)^38^. Traditional studies investigating associations between reactogenicity and immunogenicity have relied on easily accessible measures such as pain, swelling, or fever. However, these are subjective, difficult to quantify, or may depend on the vaccine administration technique^39^. Temperature measurement provides an attractive, objective, and quantifiable alternative. Prior work using infrared imaging similarly reported heterogeneous thermal responses, yet without evaluating their immunological consequences^40^. They reported a mean temperature increase of +0.70 °C at 24 h post-vaccination, with 40% experiencing a thermal reaction of at least +1 °C. Our in vitro data further suggest that immune cells themselves may contribute to local heat production, potentially via mitochondrial uncoupling. Mitochondria physiologically maintain temperatures that exceed core body temperature, which may reach 50 °C in activated mitochondria^41^. The relevance of these mechanisms to vaccine reactogenicity remains to be further defined.

The observation that pre-existing immunity shapes the adaptive vaccine response is not novel. The concept of antibody feedback summarizes the interference of pre-existing antigen-specific antibodies with the subsequent evolution of adaptive immunity upon revaccination or reinfection^6^. Thereby, immune complexes can enhance antigen uptake and presentation, potentially resulting in a more efficient antigen presentation and a more robust adaptive immune response^42^. However, antigen uptake in macrophages may limit antigen availability for B cell activation and sustained germinal center engagement. Similarly, pre-existing antibodies may mask epitopes, potentially promoting high-affinity B cell responses^43^, select for B cell responses against subdominant or less conserved epitopes (32), or prevent antigen uptake or B cell stimulation through interference^13,44,45^. Our data does not distinguish between these non-mutually exclusive mechanism.

The HA-IgG-IC associated enhanced FcR-mediated innate activation, including monocyte activation, shapes the inflammatory milieu at the vaccination site. Strong innate inflammation associated with a features of an extrafollicular B cell response in our clinical cohort. Specifically, we observed a pronounced early plasmablast response at day 7 post-immunization and lower-avidity HA-specific IgG at day 28, in relation to strong thermal reactions. In this context, elevated local temperature may act as a modulatory factor rather than a primary driver, potentially influencing cellular metabolism, activation kinetics, or antigen processing during early immune response events. As a complementary explanation, accelerated extrafollicular responses may be fueled by preferential activation of memory B cells primed from prior influenza exposures, potentially further limiting the amount of antigen available for prolonged germinal center reactions and de novo affinity maturation. Future research should evaluate the baseline level of HA-specific memory B cells to explore their role in competing for and scavenging antigens. Including BCR sequencing data will help determine whether germinal center reactions are altered in individuals with strong local thermal reactions. Finally, vaccine antigen-IgG immunocomplexes increase the size and valency of the vaccine antigen. High-valency antigens induce a low-affinity, extrafollicular response, whereas smaller, low-valency antigens activate the high-affinity B cells preferentially^46^. Collectively, these mechanisms may explain the observed phenotype of extrafollicular B cell responses in those with a strong local temperature reaction and strong IC activation.

Studies with IC-based vaccine strategies and Fc-engineered HA-Fc constructs have demonstrated that antibody glycosylation and Fc configuration can profoundly influence B cell selection, raising the possibility that qualitative features of pre-existing antibodies, not only their abundance, modulate vaccine outcomes. HA-IgG-IC have been explored as potential vaccine constructs in preclinical models and in humans^47–49^. In vivo, human influenza vaccine efficacy correlated with the amount of sialylated Fc on anti-HA-IgG produced during the early plasmablast response. The authors demonstrated that this improved humoral vaccine response occurs through co-engagement of the sialylated Fc region with CD23, which triggers the inhibitory FcγRIIB. This process raises the antigenic threshold and favors the selection of high-affinity B cells cells^50^. In preclinical studies, ICs consisting of the influenza vaccine and sialylated Fc anti-HA-IgG were used to immunize mice. IC vaccination elicited higher-avidity antibody responses than HA alone, with qualitatively enhanced breadth and potency against influenza viruses^51^. A modified approach to harnessing IC in influenza vaccination is fusing HA to an Fc fragment. Immunization with these fusion proteins induced broad immune responses and high cross-clade protection in mice^52^. Combined, this suggests that not only the amount of pre-existing IgG, but also the glycosylation of the Fc part, which critically informs the effector function of the IgGs, are important to consider.

Our study’s strengths include the integration of in vivo and in vitro systems and the detailed temporal profiling, which allows us to unravel associations between immune features and the reactogenicity phenotype. We investigated immune complex-mediated immune activation in mechanistic detail and demonstrated that immune cells may produce heat in vitro in a multi-modal approach. Limitations include the relatively small cohort size, absence of a validation cohort, the exploratory nature of some in vitro analyses, and female predominance among the ΔT^hi^ subjects. We, therefore, cannot exclude that sex, hormonal differences, or relatively higher vaccine doses (per body mass compared to men) contributed to the observed phenotype. Notably, many immune genes are located on the X chromosome, including the innate sensor TLR7^53^ and the literature consistently reports higher local reactogenicity and immunogenicity to the inactivated influenza vaccine in females (reviewed in ref. ^54^). In contrast, systemic reactions occur with similar frequencies. Sex-related differences in hormones, skin thickness, blood flow, and the nervous system’s structure may promote the development of injection-site inflammation in females^54^.

Experimental limitations include that we only assessed immune complex formation and activation indirectly in vitro, as we did not perform muscle biopsies. Similarly, since direct assessment of neutrophil recruitment requires tissue sampling or whole-blood analysis, we focused on monocytes as an innate cell subset. Histopathological studies on local vaccine reactogenicity have been done mostly for specific vaccines. In humans, a combined PET/CT and needle biopsy study for transcriptomics investigated gene signatures following intramuscular immunization with an adjuvanted, but not the standard, influenza vaccine^55^. Mouse studies compared histopathologic findings one month post-immunization with various vaccines, including the influenza vaccine. The timing of sampling and the absence of pre-existing immunity in this animal study preclude conclusions regarding immune complex formation^56^. Whether it is appropriate to perform muscle biopsies in human volunteers following a seasonal influenza vaccine for research purposes is debatable. Animal models with pre-existing pathogen-specific immunity could be employed to study local immune complex formation and complement activation in the future.

Finally, we cannot exclude that affinity maturation in subjects with strong local reactogenicity follows a different trajectory that was missed due to the single later timepoint at 28 days post-immunization. Studies in humans have shown that following repeated immunization with the flu vaccine, affinity maturation is limited and often completed within 4 weeks^57,58^ but can extend over months^59^.

In conclusion, our study identifies an association between pre-existing humoral immunity, local thermal reactogenicity, and qualitative features of the antibody response following standard, non-adjuvanted influenza protein vaccination. Our findings suggest that the consequences of vaccine reactogenicity may extend beyond antibody magnitude to include qualitative aspects of the humoral response. Further research is necessary to determine whether adjusting the thermal response at the injection site or via metabolic interventions can improve vaccine efficacy by balancing reactogenicity and immunogenicity. Conducting a clinical study to assess if non-steroidal anti-inflammatory drugs (NSAIDs) influence local thermal reactogenicity and subsequent immune responses would be relatively simple. Interestingly, in pediatric studies, NSAIDs did not significantly affect antibody responses to various vaccines^60,61^, although none of these studies tested antibody avidity or HAI titers as functional outcomes. Understanding how local inflammation and IC-driven pathways modulate vaccine immunogenicity may inform strategies to optimize antigen design, Fc engineering, and formulation to enhance protective immunity while limiting unwanted reactogenicity.

## Methods

### Study participants

The local ethics committee (Ethics Commission of North-Western and Central Switzerland, EKNZ #2017-01726) approved the study (ARIVA-Study; NCT04059991, registration date on ClinicalTrials.gov: 2017-11-01) that was conducted in accordance with the Declaration of Helsinki. All subjects were healthy, provided written informed consent, and received no other vaccinations during the study. None of the study participants reported use of non-steroidal anti-inflammatory drugs (NSAIDs) during the 24 h after the vaccination, or was taking corticosteroids, biologics, or other immunomodulatory medications. The majority of participants were healthcare workers or laboratory personnel. All subjects were vaccinated between November and December 2019 with the same inactivated, non-adjuvanted, quadrivalent influenza split vaccine (Vaxigrip Tetra, Sanofi 2019/20), containing 15 μg hemagglutinin per strain (A/Brisbane/02/2018; A/Kansas/14/2017; B/Colorado/06/2017; and B/Phuket/3073/2013) in a total volume of 0.5 mL. We gathered data through a questionnaire on self-reported influenza-like illness over the last five years, the total number of influenza vaccines received during this period, personal histories of allergic diseases and autoimmunity, and demographic details including sex, BMI, and age.

### Assessment of reactogenicity and local thermal reactions

We utilized a contact-free temporal thermometer (Withings, Europe) for all temperature assessments. According to the company, this thermometer employs 16 infrared sensors to take over 4000 measurements within seconds (HotSpot Sensor Technology). It operates within a range of 35 °C–43.2 °C (95 °F–109.8 °F), featuring a resolution of 0.1°C (0.2 °F and a clinical accuracy of ±0.2°C (±0.4 °F) for measurements in the temporal area. We measured the temperature at the vaccine injection site (deltoid region) immediately before and 24 h after vaccination, as well as at the same location on the opposite arm and both temporal regions (i.e., systemic temperature) for comparison. Measurements were conducted in duplicate, and average values were calculated to determine the temperature difference before and after vaccination at the injection site [ΔT (24h–0h)], between the vaccinated and control arm [ΔT(vaccine) − (control)], or systemically. The ‘local thermal response’ was defined as the ΔT at the vaccination arm. In addition, study participants subjectively rated their pain 24 h post-vaccination as: none, mild, or strong. We examined the vaccination site for erythema (redness) and, if present, measured the largest diameter in centimeters (cm).

### Sampling and peripheral blood mononuclear cell (PBMC) isolation

We collected serum and ethylenediaminetetraacetic acid (EDTA) blood samples before, after 24 h, and on days 7 and 28 post-vaccination for immunological analyses. To isolate the PBMC fraction, the EDTA blood was diluted in PBS and layered over 16.5 ml Lymphoprep medium (density 1.077 g/ml) in centrifuge Leucosep tubes (Grainer Bio-One, Austria). PBMCs were isolated by standard Ficoll-Paque density-gradient centrifugation at 700 × *g* for 15 min with a low acceleration and no breaks^62^. The enriched PBMC layer was collected and washed twice in PBS, and cells were used immediately or cryopreserved in FBS containing 10% DMSO until further use.

### Elispot to measure influenza-specific T cell responses

Standard IFN-γ Elispot was performed using 150,000 PBMC/well. We stimulated the cells with 1μg/ml recombinant hemagglutinin (A/Brisbane/02/2018 (H1N1) and A/Kansas/14/2017 (H3N2)) (both from (eENZYME LLC, Gaithersburg, MD, USA) or with the commercial influenza vaccine (QIV; Vaxigrip Tetra, Sanofi 2019/20) in a 1:200 dilution on a IFNγ-coated PVDF (polyvinylidene fluoride) plate (Mabtech, Nacka Strand, Sweden) at 37 °C, 5% CO_2_ for 18 h. 0.5 μg/mL SEB (staphylococcal enterotoxin B) for polyclonal T cell activation or media alone served as controls. Elispot data were expressed as spot-forming cells per million PBMC (SFU/M).

### Measurement of the influenza-specific antibodies by multiplexed Luminex assay

Using a custom-made Luminex assay, we quantified antibodies specific to the H1 and H3 vaccine strain. Different Luminex MaxPlex®beads were coated with recombinant hemagglutinin (HA) from the vaccine strains A/Brisbane/02/2018 (H1N1) and A/Kansas/14/2017 (H3N2) according to the manufacturer’s instructions. Briefly, 1.25 μg of HA protein was coupled onto 1.25 million beads in 500 μl of 50 mM MES buffer (pH 5.0) for 2 h at RT with constant rotation. The bead coupling to different HA was verified using an anti-His antibody (1 μg/mL, A00174, GenScript) and a PE-labeled anti-rabbit IgG secondary antibody (1 μg/mL, 406421, BioLegend) that detects the his-tag on the HA. To measure HA-specific serum IgG, we incubated 1000 beads with sera diluted 1:100 and following the manufacturer’s instructions^63^. We used 1 μg/mL of the respective mouse anti-human detection antibodies to quantify Influenza-specific IgM (SA-DA4), IgG (JDC-10), IgG1-4 subclasses (HP6001, HP6002, HP6050, HP6025) and IgA (2053-01; all from SouthernBiotech, Birmingham, AL, USA). Bovine serum albumin (BSA)-coated beads were the negative control. Data was acquired as MFI on a Luminex 200 reader. To compare subclass-specific responses between groups, we calculated relative responses per immunoglobulin class and antigen, i.e., median MFI ΔT^hi^ divided by MFI ΔT^lo^.

### Hemagglutination inhibition assay

We measured influenza strain-specific hemagglutination inhibition (HAI) titers against A/Brisbane/02/2018 (H1N1) and A/Kansas/14/2017 (H3N2) (NIBSC, Hertfordshire, UK)^64^. The influenza virus concentration was adjusted to four hemagglutination units (HAU) in a standard hemagglutination assay. Pre- and post-vaccination sera were treated with a receptor-destroying enzyme (Denka Seiken Co., Ltd, Tokyo, Japan) for 18 h at 37 °C, followed by heat-inactivation at 56 °C for 1 h. Samples were diluted two-fold serially in PBS in duplicates and mixed 1:1 with the virus. After one hour at 37 °C, formaldehyde-fixed guinea pig red blood cells (1.5%) were added and incubated for 1 h at 4 °C^64^. The HAI titer was defined as the highest serum dilution that inhibited hemagglutination.

### Hemagglutinin-IgG immune complex formation, C1q binding assay and in vitro complement activation

Hemagglutinin-IgG immune complexes (HA-IgG-IC) were generated by mixing the recombinant HA at 0.5 μg/mL HA (HA used as described above) with participants’ sera at a 1:10 dilution. Complement binding capacity of the HA-IgG-IC was assessed using the QUANTA Lite C1q CIC ELISA (Inova Diagnostics/Werfen, Barcelona, Spain) following the manufacturer’s instructions. Briefly, HA-IgG-IC were incubated in C1q-coated microplate wells, and C1q binding of the IC was detected using a horseradish peroxidase–conjugated goat anti-human IgG antibody and quantified by measuring absorbance at 450 nm. Immune complex concentrations were extrapolated from the calibration curve and expressed as heat-aggregated human IgG equivalents per mL (μg Eq/mL).

To assess in vitro complement activation, we coated plates with 5 µg/mL C1q protein (Complement Technology Inc., Tyler, TX, USA) in 0.2 M carbonate buffer (pH 9.6) overnight at 4 °C, blocked with PBS + 1% BSA. Serum samples were diluted 1:50 in either HBS (HEPES-buffered saline) or PBS supplemented with 10 mM EDTA and incubated on the plates for 1 h at 37 °C with shaking at 300 rpm. Complement activation was assessed by detecting C4d deposition using rabbit anti-human C4d (Abcam, Cambridge, UK, 1:400) followed by HRP-conjugated donkey anti-rabbit IgG (BioLegend, San Diego, CA, USA, 1:4000) and TMB substrate (Invitrogen, Waltham, MA, USA), and absorbance was measured at 450 nm^28^.

### Antibody avidity determination using Biolayer Interferometry (BLI) and urea challenge

To assess anti-HA-antibody avidity by biolayer interferometry, 10 μg/mL His-tagged hemagglutinin (A/Brisbane/02/2018 (H1N1) and A/Kansas/14/2017 (H3N2)) was loaded onto Ni-NTA biosensors (Sartorius, Göttingen, Germany) in running buffer (PBS, 0.02% Tween-20, 0.1% BSA) for 300 s. Sensors were dipped in running buffer for 60 s, then into serum-containing buffer for association (300 s), followed by dissociation in buffer for 600 s. Regeneration involved three cycles of 20 mM glycine in PBS, alternating with running buffer, followed by reactivation in 20 mM NiCl₂ for 120 s. All steps were performed at 1000 rpm. KD values were calculated using Octet® CFR software (ForteBio, Fremont, CA, USA). In select experiments, IgG avidity was assessed in addition (or solely) by urea challenge, comparing Luminex MFI values of HA-specific IgG after 30 min incubation with either PBS or 6 M urea (room temperature, 800 rpm). The avidity index (AI) was defined as [MFI with incubation in urea solution] / [MFI with incubation in PBS].

### Monocyte phenotyping, in vitro maturation and antibody-dependent cellular phagocytosis assay

Primary monocytes were isolated from PBMC taken at the pre-vaccination timepoint from subjects with low and high local temperature reactions. Monocyte subsets were sorted on a FACSAria® III (BD Biosciences) using CD3 (Alexa Fluor 700, OKT3), CD19 (Alexa Fluor 488, HIB19), CD14 (FITC, 63D3), CD16 (BUV 496, 3G8), and a viability dye (eFluorTM 780, Invitrogen^TM^). Sorted monocytes or the monocyte cell line THP-1 (ATCC® TIB-202^™^) were differentiated into monocyte-derived dendritic (mDC) cells for five days in the presence of 1000 U/ml GM-CSF and IL-4 each, replenished at days 2 and 4. For mDC maturation, 200 pg/ml LPS was added for 24 h. Ex vivo monocytes and mDCs were incubated with fluorochrome-coupled hemagglutinin in the presence or absence of sera (1:10) from subjects with low- or high-temperature reactions, for 4 h. Fluorochrome-coupling of the H3N2 A/Kansas/14/2017 hemagglutinin (10 µg/reaction) was performed using the Lightning-Link® Allophycocyanin (APC) conjugation kit (Expedeon, San Diego, CA, USA) according to the manufacturer’s instructions. Briefly, 10 μg hemagglutinin was mixed with the Modifier reagent at a ratio of 1:10. The antibody-Modifier mixture was added directly to a vial containing lyophilized fluorochrome conjugation mix (APC) and resuspended by gentle pipetting. The reaction was incubated for 3 h at room temperature in the dark. Following incubation, Quencher reagent was added at a 1:10 ratio, gently mixed and incubated for 30 min at room temperature. The resulting conjugated antibody was ready for use without further purification. Activation and HA uptake were assessed by flow cytometry. Antibodies used to characterize the monocyte activation profile are listed in Supplementary Table 1. A viability dye (eFluorTM 780, Invitrogen^TM^) was included in all experiments.

In vitro cytokine production was quantified in the culture supernatants of monocytes activated as indicated in the respective experiments. We used ELISA kits for IL-1 and IL-6 (Peprotech, Cranbury, NJ, USA) according to the manufacturer’s instructions. For the Fc-receptor blocking experiments, we used antibodies against FcγR1 (CD64, 10.1) [10 μg/ml]), FcγR2 (CD32, FUN-2) [10 μg/ml]), and FcγR3 (CD16, 3GB) [10 μg/ml]) (all from BioLegend). THP-1 monocytes were preincubated with blocking antibodies for one hour before adding fluorochrome-labeled hemagglutinin and serum. The effect of blocking was calculated as percent inhibition ((H3 uptake without blocking/H3 uptake with the Fc block) × 100). After four hours of incubation, cells were stained using antibodies listed in Supplementary Table 1 and acquired on a BD LSRFortessa. Analysis was performed using FlowJo v.20.6.2.

### In vitro temperature measurements of cellular heat production

THP-1, PBMCs, or sorted cell subsets were resuspended in complete RPMI medium and transferred into a DeepWell 96 U well plate. After an acclimatization period of 1 h at 37 °C, the cells were stimulated with HA-IgG-IC, LPS [1 ng/ml, for monocyte activation], CpG [1 μg/ml, for B cell activation], anti-CD3/CD28 antibodies [2 μg/ml, for T cell activation], PMA [0.1 μg/ml, for NK cell activation] or maintained in the medium as a control. The temperature of the culture medium was recorded every minute during the acclimatization period and a three-hour stimulation using a data logger thermometer (SEFRAM9814 Datenlogger-Thermometer) with a liquid-compatible immersion probe (TC type K. Testo). The average temperature for the entire three-hour period was summarized in bar graphs.

### Mitochondrial temperature production assay using MitoThermo Yellow (MTY)

THP-1 or human primary monocytes were stimulated with the quadrivalent influenza vaccine [1:200] or LPS [1 ng/ml] for six hours at 37 °C, 5% CO_2_. Cells were then stained with 100 nM MTY, 100 nM MitoTracker Green (Invitrogen, Waltham, MA, USA), or both for 20 min at 37 °C and 5% CO_2_. After washing, the cells were fixed and permeabilized with the BD fixation and permeabilization solution, and counterstained with DAPI (Biolegend, San Diego, CA, USA). Cells were mounted with Vectashield fluorescence mounting medium (Vector Laboratories, Burlingame, CA, USA) and analyzed using the Nikon A1 confocal microscope with a 100x oil-immersion objective (Nikon Plan Apo 100×1.45NA oil). Stacked images were acquired at 0.1 mm intervals throughout the cell body. The fluorescence intensity of MTY overlapping with MitoTracker staining was quantified using ImageJ (Fiji, v.2.9.0). Data are expressed as MFI.

### Temperature effects on B cell function in vitro

To study the direct effects of higher temperature on immune cells, specifically B cells, we performed experiments at different cell culture temperatures. To this end, we set the incubator temperature set-point to 37.5 °C or 39 °C. The 39 °C was chosen to approximate the elevated tissue temperature at the vaccination site. The estimate was based on the observed increase in skin surface temperature. The readouts focused on B-cell functions. PBMCs from the pre-vaccination time point were stimulated with the influenza vaccine (Vaxigrip®) [1/200] or a combination of CD40L [100 ng/ml] and IL-21 [100 ng/ml] at 37 °C or 39 °C. To assess proliferation, PBMCs were stained with the CellTrace™ Violet Cell Proliferation Kit (Thermo Fisher Scientific, Waltham, Massachusetts, USA) according to the manufacturer’s instructions before stimulation. On day five, proliferation was quantified as %CTV low B cells among total B cells and plasmablast generation was assessed using antibodies listed in Supplementary Table 1 and a viability dye (eFluor^TM^ 780, Invitrogen^TM^). All flow cytometric experiments were acquired on an LSRFortessa (BD Biosciences, San Jose, CA) and analyzed using FlowJo (v.10.6.2). Total antibody production in the cultures was assessed after 7 days using an IgG and IgM ELISA (Invitrogen, Waltham, Massachusetts, USA). The influenza-specific IgG levels were measured using the multiplexed Luminex assay as described.

### Direct ex vivo immune cell phenotyping

Peripheral blood mononuclear cells (PBMC) were stained immediately following isolation (as described above) for immuophenotyping. The antibodies used for in vivo T cell, B cell, and Monocyte phenotyping are listed in Supplementary Table 2. A viability dye (eFluor^TM^ 780, Invitrogen^TM^) was included in all panels. All samples were acquired on a BD LSRFortessa (BD Biosciences, San Jose, CA) and analyzed using FlowJo v.10.9.0. Gating strategies are indicated in the Supplementary Figs. 1 and 2.

### Data analysis and statistics

Group comparisons were performed using non-parametric, two-tailed *t*-tests (Mann-Whitney). We considered *p*-values < 0.05 statistically significant and reported the detailed *p*-values in the results and figure legends. Correlations of continuous variables were assessed using the non-parametric Spearman’s rank correlation. Comparisons of more than two groups were done using one-way ANOVA and Dunn’s test. Data was visualized using GraphPad Prism (Version 10.4.2).

## Supplementary information

44400_2026_95_MOESM1_ESM

## Data Availability

All data will be made available by the authors on reasonable request.
