# Supplementary material for: Preexisting IgG forms immune complexes and links local thermal reactogenicity with immunogenicity in influenza vaccination
Source: NPJ Vaccines. 2026 May 2;11:137. doi: 10.1038/s41541-026-01477-x (PMC13342333; doi:10.1038/s41541-026-01477-x)
Supplement: Supplementary file 1 — 44400_2026_95_MOESM1_ESM [file 41541_2026_1477_MOESM1_ESM.pdf]

Supplementary Material

Pre-existing hemagglutinin specific IgG forms immune complexes and links local thermal reactogenicity with immunogenicity in seasonal influenza vaccination

Julia R. Hirsiger et al.

__Content__

Supplementary Figure 1: Gating strategy for B cell subsets.

Supplementary Figure 2: Gating strategy for T cell subsets.

Supplementary Figure 3: Correlation between erythema and local thermal reactions.

Supplementary Figure 4: Pre-vaccination immunophenotyping of monocytes, T, and B cells in ΔT^{hi} vs. ΔT^{lo} subjects.

Supplementary Figure 5: Baseline influenza-specific immunoglobulin subclass responses.

Supplementary Figure 6: Unsupervised cluster analysis of baseline characteristics and the local thermal reaction.

Supplementary Figure 7: Hemagglutinin-serum immunocomplex phagocytosis is antibody-mediated.

Supplementary ***Figure 8: Hemaglutinin-strain-specific monocyte activation and ADCP stratified by the local thermal reaction.***

Supplementary Figure 9: Unsupervised cluster analysis of baseline immune profiles and innate effector functions.

Supplementary Figure 10: Correlates ADCP and monocyte activation.

Supplementary Figure 11: Monocyte activation in vivo post-immunization and in vitro heat production.

Supplementary Figure 12: Temperature-dependent IgM secretion.

Supplementary Figure 13: Subclass resolved hemagglutinin-specific antibody response.

Supplementary Figure 14: Correlations of local thermal reactions and adaptive vaccine response.

Supplementary Table 1: Antibodies used in in vitro experiments

Supplementary Table 2: Antibodies used for in vivo phenotyping

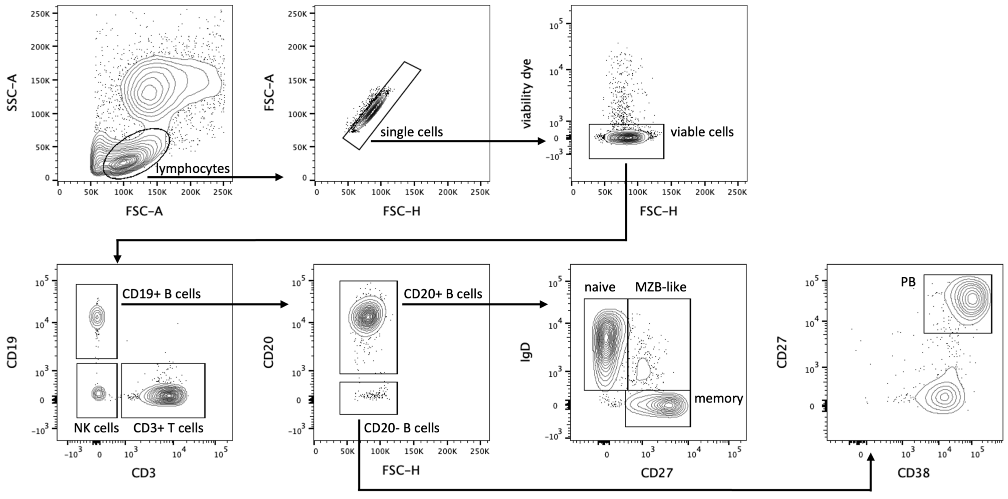

Supplementary Figure 1: Gating strategy for B cell subsets. B cells were identified as single, viable CD19+CD3- lymphocytes. CD20+ B cells were further subdivided into naïve (CD27- IgD+), memory (CD27+ IgD-), and marginal zone-like B cells (MZB) (CD27+ IgD+). Plasmablasts were identified as CD20-CD27+CD38high.

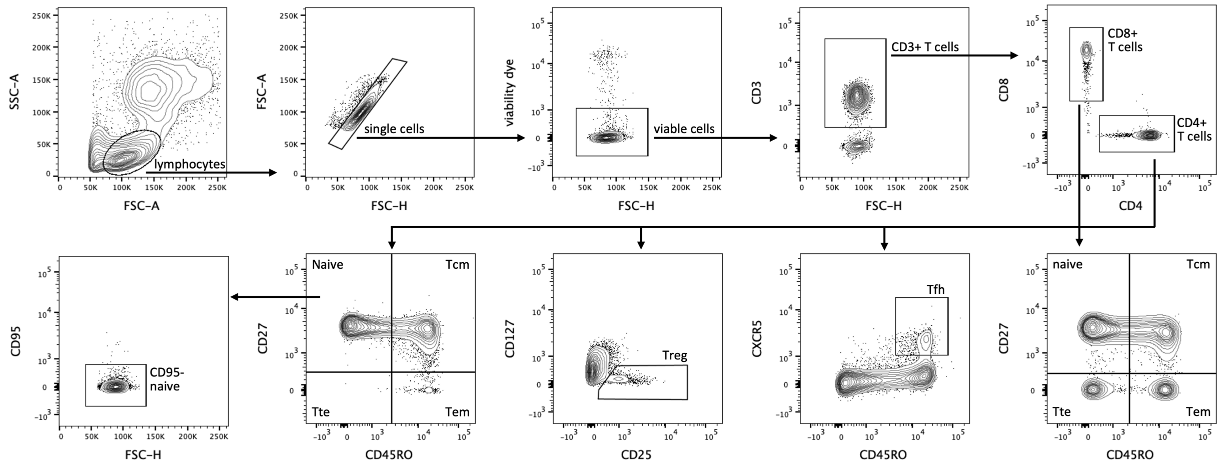

Supplementary Figure 2: Gating strategy for T cell subsets. T cells were identified as single, viable CD3+ lymphocytes. CD8 and CD4 T cells were subdivided into naïve (CD45RO-CD27+CD95-), central memory (CD45RO+CD27+), effector memory (CD45RO+CD27-) and terminal effector (CD45RO-CD27-) T cells. CD4 T cells were additionally characterized as CD127low/-CD25high Treg and CD45RO+ CXCR5+ T follicular helper cells (Tfh).

A

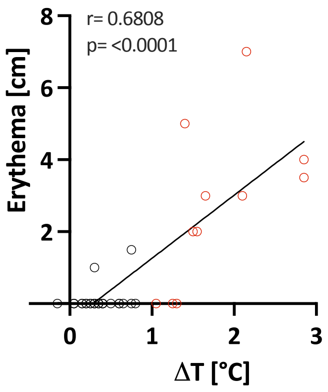

Supplementary Figure 3: Correlation between erythema and local thermal reactions. Correlation analyses between the local temperature difference (ΔT= T(vaccine) – T(control) arm at 24h post-vaccination) vs. the erythema size (in cm) 24 h post-immunization. Correlation analyses were performed using simple linear regression; r and p values were calculated using Pearson's correlation (n = 38).

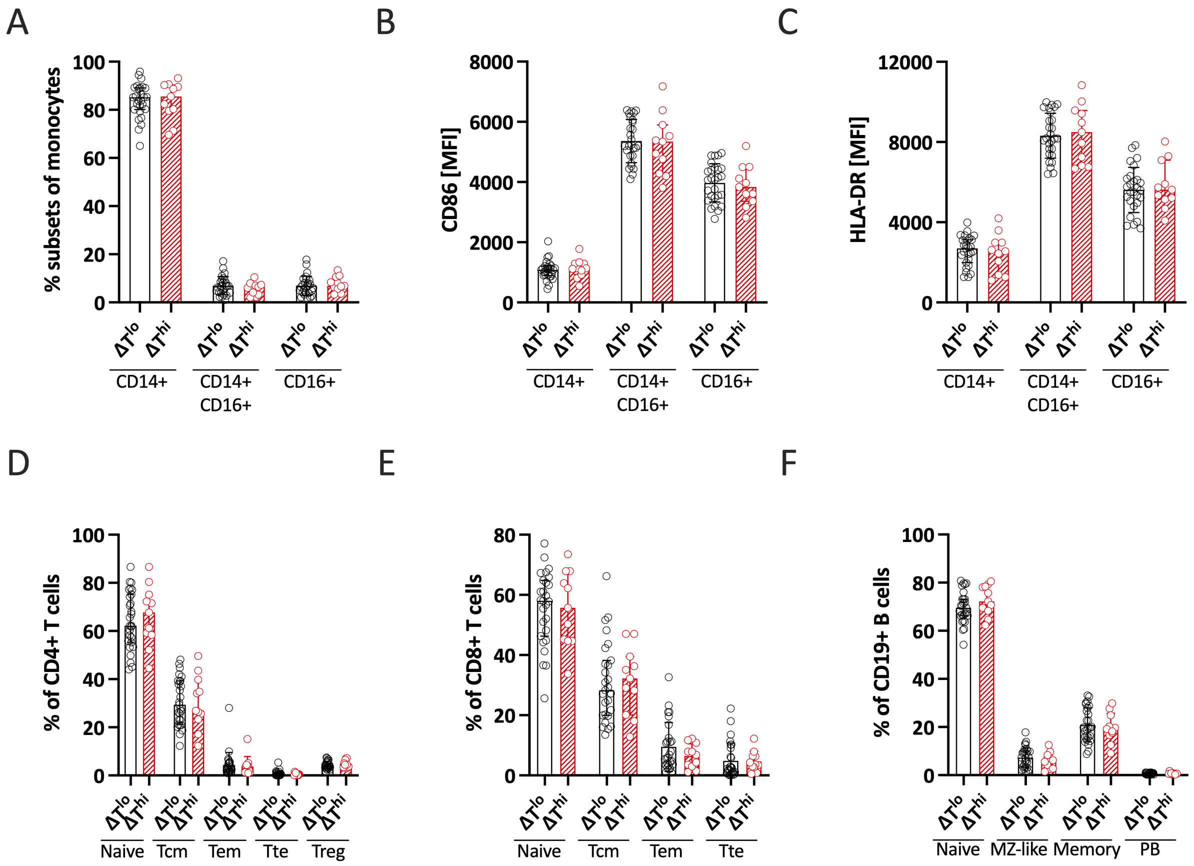

Supplementary Figure 4: Pre-vaccination immunophenotyping of monocytes, T, and B cells in ΔT^{hi} vs. ΔT^{lo} subjects. (A) Frequency of classical (CD14^{high}CD16^{negative}), intermediate (CD14^{high}CD16^{low}), and non-classical (CD14^{low}CD16^{high}) monocytes. (B) The activation state of the monocyte subsets was assessed by measuring CD86 and (C) HLA-DR expression. (D) CD4 phenotyping in naïve (Tn), central memory (Tcm), effector memory (Tem), terminal effector (Tte), and regulatory (Treg)T cells. (E) CD8 phenotyping in naïve (Tn), central memory (Tcm), effector memory (Tem), and terminal effector (Tte) T cells. (F) B cell phenotyping in naïve, marginal zone-like (MZ-like), memory B cells, and plasmablasts (PB). Each symbol represents an individual donor (n=27 ΔT^{lo}, n=11 ΔT^{hi}). Data are presented as median ± IQR (A-F).

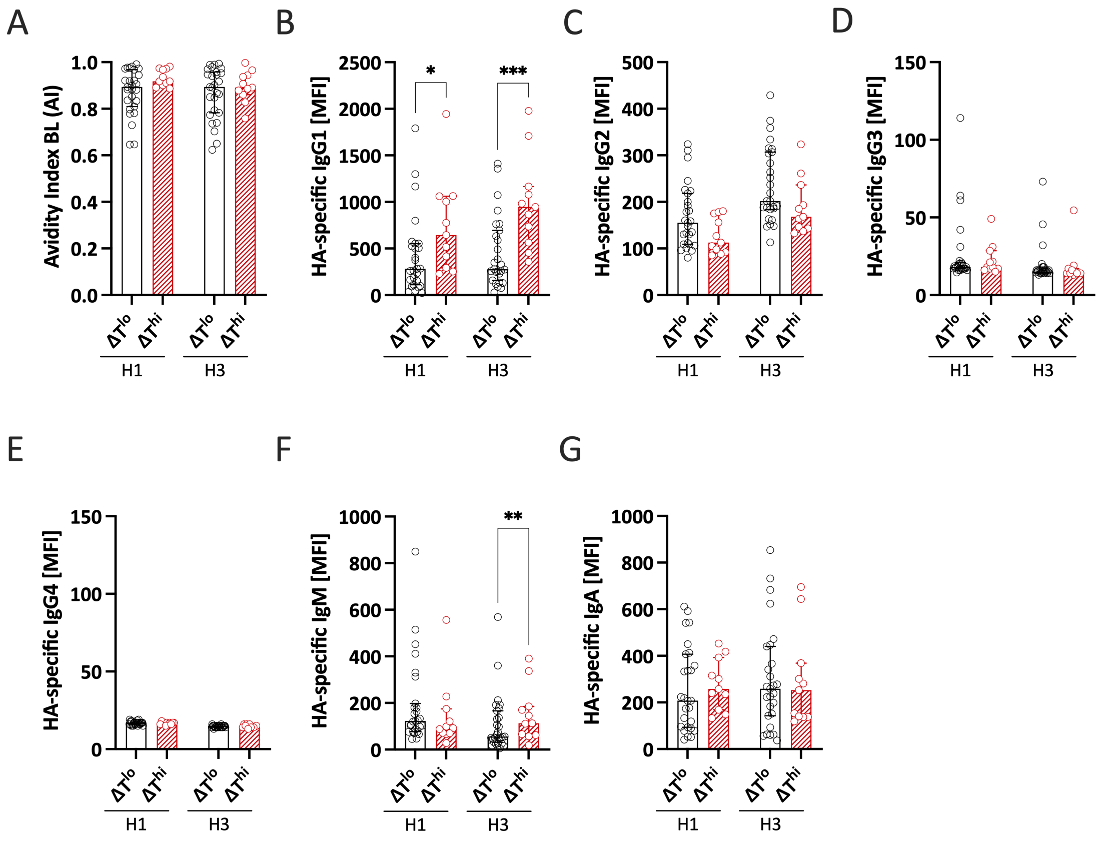

Supplementary Figure 5: **Baseline influenza-specific immunoglobulin subclass responses.** (A) Avidity index (urea challenge) of the total H1- and H3-specific IgG pre-immunization. (B-G) Absolute MFI values of the influenza-H1- and H3-specific IgG1-4 subclasses, IgA, and IgM pre-vaccination are shown for ΔT^{hi} vs. ΔT^{lo}. Data represent the raw data used for the ratios shown in Figure 2E. Each symbol represents an individual donor (n=27 ΔT^{lo}, n=11 ΔT^{hi}). Data are presented as median ± IQR (A-G). Statistical comparisons were performed using Mann-Whitney U tests, **p*<0.05, ***p*<0.01, ****p*<0.001.

A

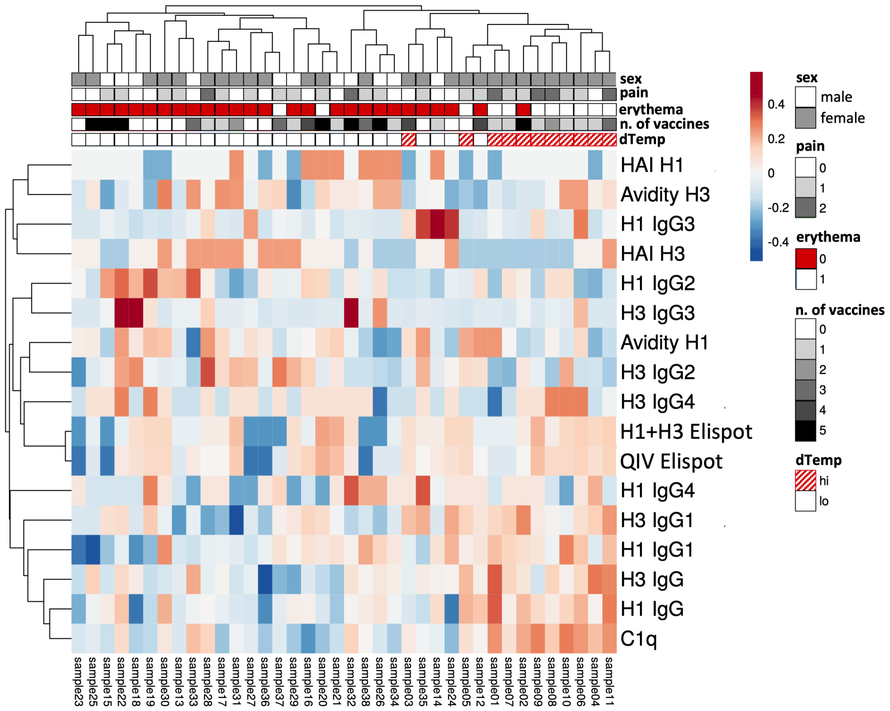

Supplementary Figure 6: **Unsupervised cluster analysis of baseline characteristics and the local thermal reaction.** Heatmap showing the baseline characteristics of the vaccine cohort (n=38). Demographic and clinical features (sex, pain score, erythema, number of vaccines received in the past 5 years, and ΔT^{hi} and ΔT^{low} as indicated on the top of the heatmap) are clustered with immunological data (pre-vaccination HA-specific IgG subclases, HAI titers, Avidity, C1q binding, and Elispot responses; indicated for H1 and H3 hemaglutinin) using the open-user Clustvis online tool. Rows were centered and scaled to unit variance without transformation. Both rows and columns were clustered using correlation distance and average linkage.

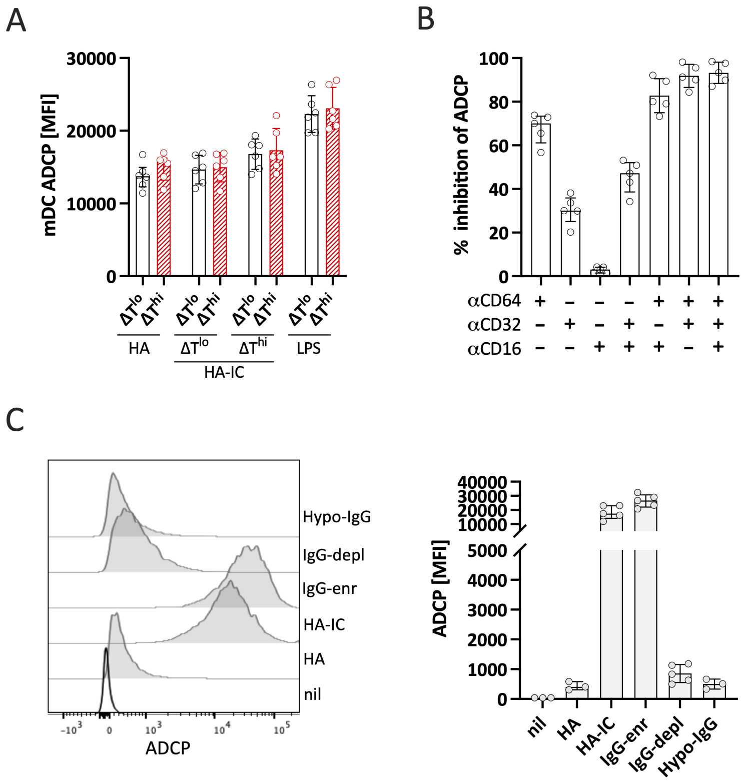

Supplementary Figure 7: **Hemagglutinin-serum immunocomplex phagocytosis is antibody-mediated.** (A) Comparison of spontaneous, ADCP-mediated, and TLR-stimulated HA phagocytosis in ΔT^{hi} vs. ΔT^{lo} monocyte-derived dendritic cells (mDC). (B) Effects of different combinations of FcγR blocking on ADCP, expressed as % inhibition in phagocytosing fluorochrome-labeled HA by THP-1. CD64= FcγR1, CD32= FcγR2, CD16= FcγR3. (C) Histogramm showing increased HA-uptake by THP-1 cell line (i.e., ADCP) in the presence of antibodies (HA-IC). In contrast, IgG-depleted serum (IgG-depl) and serum of a patient with severe hypogammaglobulinemia (Hypo-IgG) show only minimal to no HA uptake. Serum enriched (IgG-enr)for higher IgG concentrations further increased ADCP. Data are presented as median ± IQR. HA-IC ΔT^{hi} and ΔT^{lo}= immune complexes from serum and HA. LPS= lipopolysaccharide. ADCP= antibody dependent cellular phagocytosis. MFI= median fluorescence intensity. Pooled data of n= 3 independent experiments with two donors per experiment (n=6 ΔT^{lo}, n=6 ΔT^{hi}). Data are presented as median ± IQR.

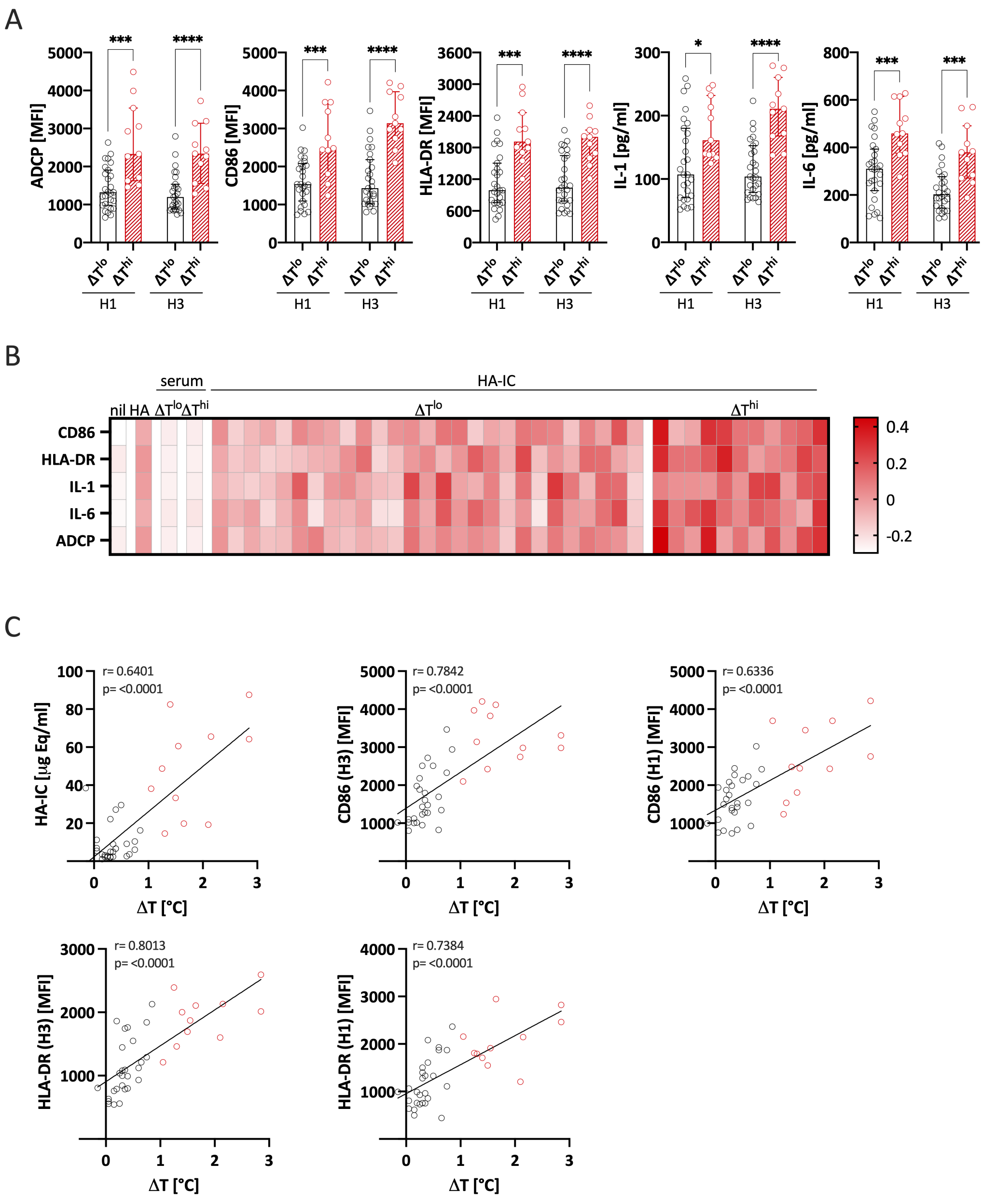

Supplementary Figure 8: **Hemaglutinin-strain specific monocyte activation and ADCP stratified by the local thermal reaction.** (A) Comparison of ADCP, monocyte activation (CD86 and HLA-DR upregulation), and cytokine secretion (IL1 and IL6) following stimulation of THP-1 cells with HA-IgG immune complexes derived from ΔT^{hi} vs. ΔT^{lo} subjects. H1 and H3 indicate which hemaglutinin was used for immune complex formation. Statistical comparisons were performed using Mann-Whitney U tests, **p*<0.05, ****p*<0.001, *****p*<0.0001. (B) Heatmap summary graph of the H1-stimulated THP-1 data for the whole cohort (n=38) (complementing the H3 heatmap in ***Figure 3***). (C) Correlations of the ΔT at the vaccinated arm with the amount of HA-IgG immune complexes and monocyte activation. Correlation analyses were performed using simple linear regression; r and p values were calculated using Pearson's correlation (n = 38).

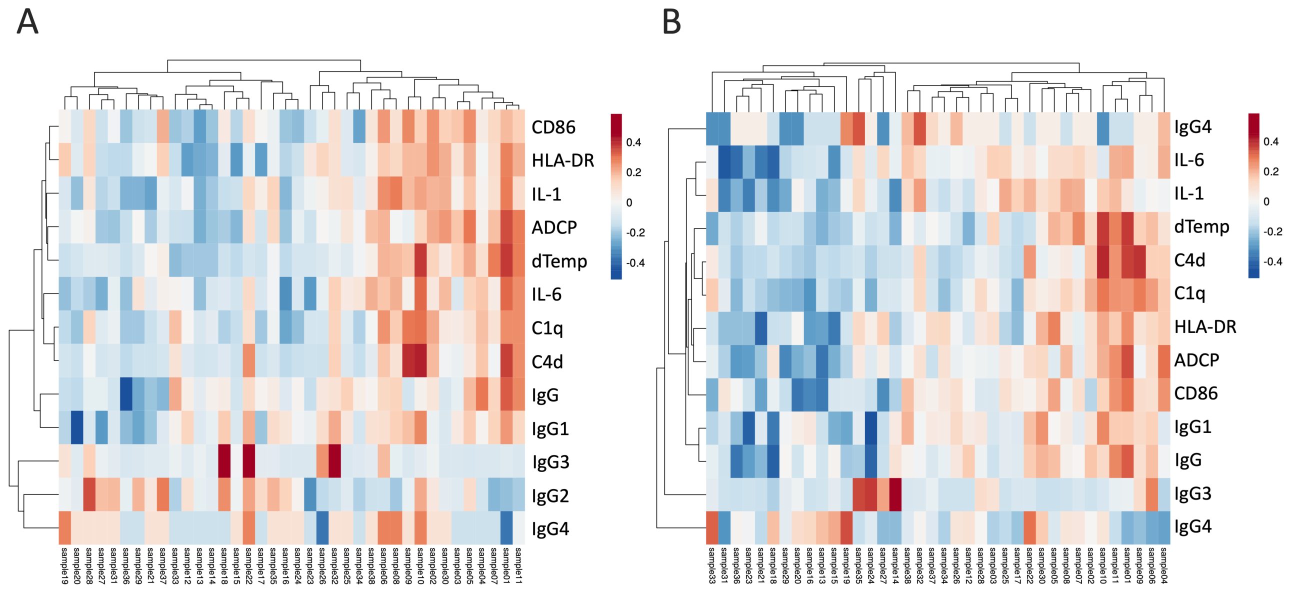

Supplementary Figure 9: **Unsupervised cluster analysis of baseline immune profiles and innate effector functions.** Pre-vaccination immunological data (pre-vaccination HA-specific IgG subclasses (IgG and IgG1-4), C1q binding of HA-IgG-IC, complement activation by HA-IgG-IC (C4d), monocyte activation (CD86 and HLA-DR), cytokine secretion (IL-1 and IL-6) and ADCP following activation by HA-IgG-IC were clustered with the absolute ΔT values. The two heat maps represent: (A) H1 hemaglutinin-specific IgG/subclasses and HA-IgG-IC; (B) H3 hemaglutinin-specific IgG/subclasses and HA-IgG-IC. The open-source Clustvis online tool was used. Rows were centered and scaled to unit variance without transformation. Both rows and columns were clustered using correlation distance and average linkage. Data shows that local thermal reactogenicity, pre-existing HA-IgG/IgG1, the amount of HA-IgG-IC and innate activation were all strongly interlinked.

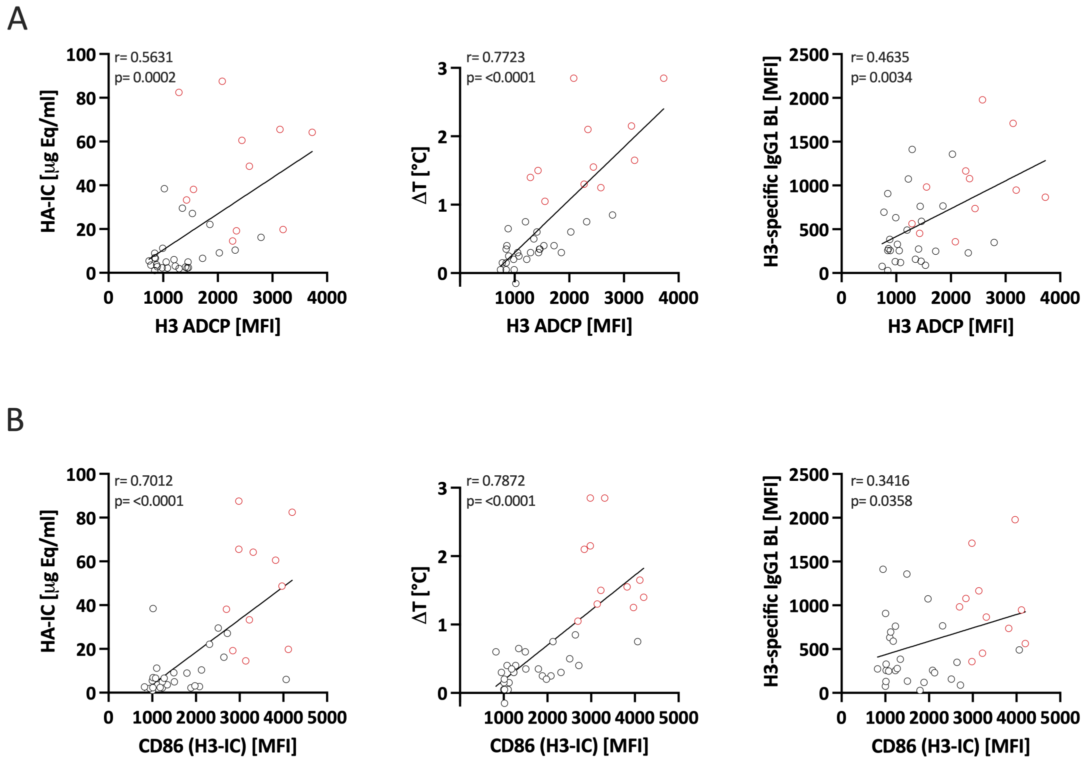

Supplementary Figure 10: **Correlates of ADCP and monocyte activation.** HA-IgG immune complexes formed from pre-vaccination serum (HA-IC), the local thermal reaction (ΔT) and pre-vaccination HA-specific IgG1 were correlated with (A) ADCP and (B) activation (CD86 upregulation) of THP-1 cells following incubation with immune complexes formed between serum from ΔT^{hi} (red open circles) and ΔT^{low} (black open circles) subjects. Correlation analyses were performed using simple linear regression; r and p values were calculated using Pearson's correlation (n = 38).

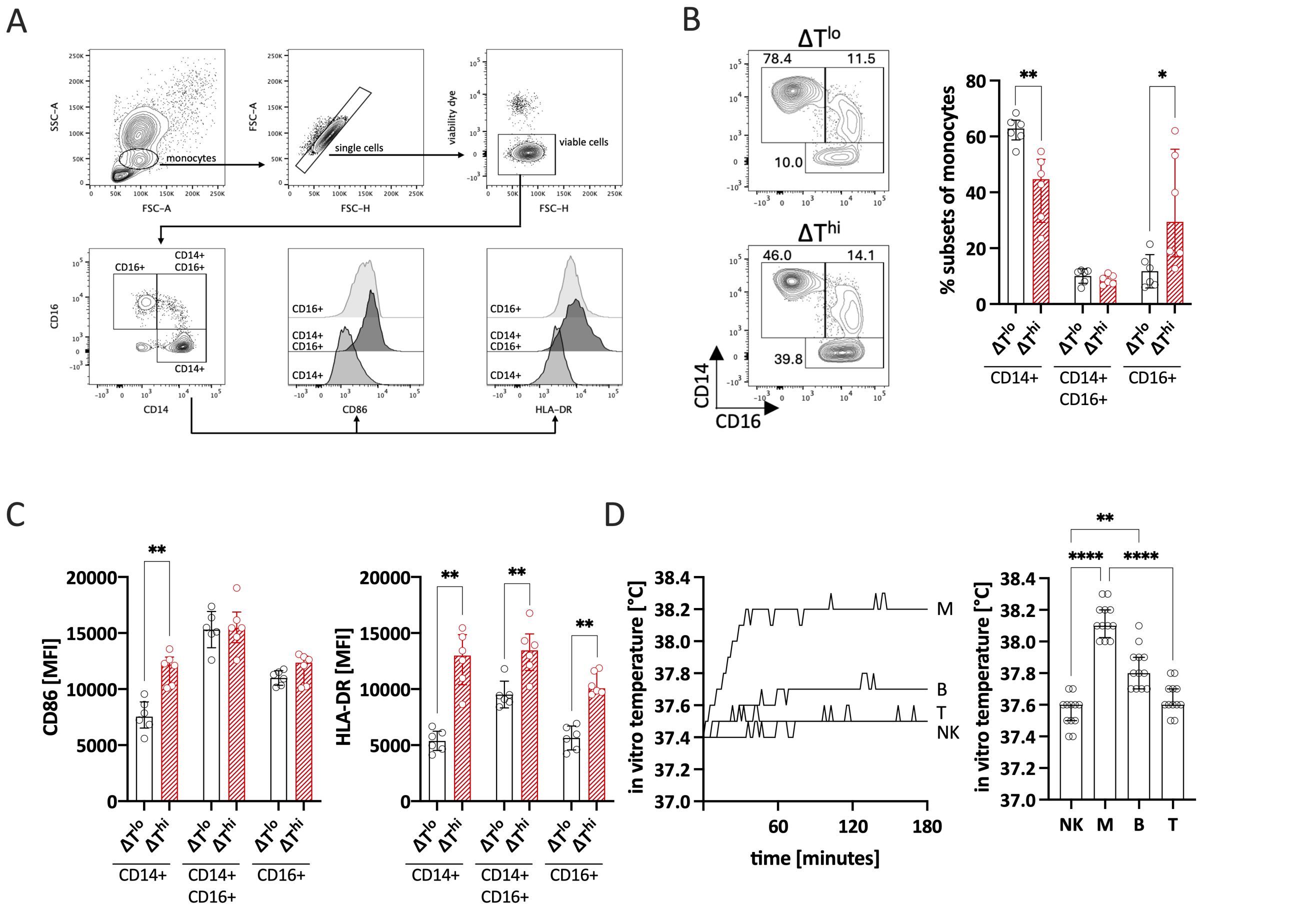

Supplementary Figure 11: **Monocyte activation *in vivo* post-immunization and *in vitro* heat production.** (A) Gating strategy for monocytes. (B) Blood samples 24h post QIV immunization were analyzed for the frequency of classical ($CD14^{high}CD16^{negative}$), intermediate ($CD14^{high}CD16^{low}$), and non-classical ($CD14^{low}CD16^{high}$) monocytes, and (C) their activation state (CD86 (left panel) and HLA-DR (right panel)). Data are presented as median ± IQR. n=6 ΔT^{hi}, n=6 ΔT^{lo}. (D) *In vitro* temperature production by sorted immune subset (M= monocytes, B= B cells, T= T cells, NK= natural killer cells) assessed over three hours after cell-subset targeted activation with anti-CD3/28 antibodies (‘T’ cells), CpG (‘B’ cells), LPS (monocytes, ‘M’), or PMA (‘NK’ cells) is shown as an example curve (left) and the summary graph (right). Representative *in vitro* temperature curve of n=6 independent experiments. Each symbol represents an individual donor (n=12). Data are presented as median ± IQR. Statistical comparisons were performed using Mann-Whitney U tests and one-way ANOVA with Dunn’s multiple comparison test as appropriate, **p*<0.05, **p<0.01, ****p<0.0001.

A

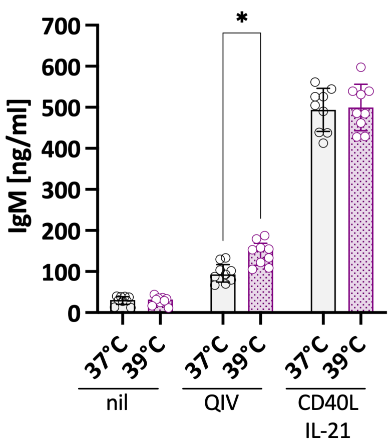

Supplementary Figure 12: **Temperature-dependent IgM secretion.** (A) Total IgM production (ELISA) of stimulated B cells was compared at 37°C vs 39°C. Data represent three independent experiments, each with three different donors (n=9). Data are presented as median ± IQR. Statistical comparisons were performed using Mann-Whitney U tests, *p<0.05.

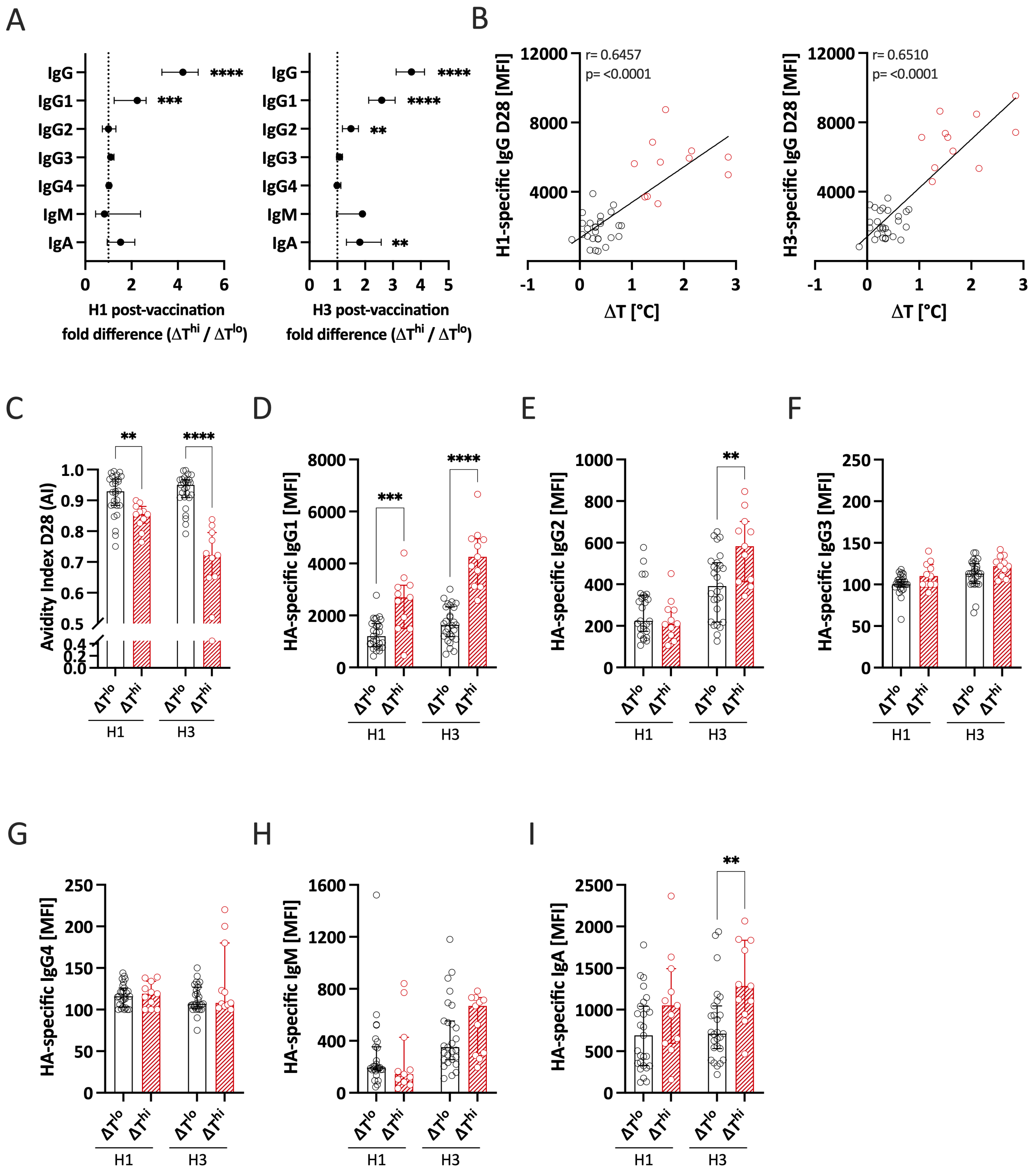

***Supplementary Figure 13*: Subclass resolved hemagglutinin-specific antibody response.** (A) Comparison of IgM, IgA, IgG, and IgG subclass response to H1 (left) and H3 (right) day 28 post-vaccination. Data expressed as the median relative response MFI ΔT^{hi} / MFI ΔT^{lo} ± upper/ lower limits. n=27 ΔT^{lo}, n=11 ΔT^{hi}. (B) The local thermal reaction correlated significantly with the d28 anti-H1 (left) and H3 IgG response (right). Correlation analyses were performed using simple linear regression; r and p values were calculated using Pearson's correlation (n = 38). (C) Avidity index of H1 and H3-specific IgG on d28 in ΔT^{hi} vs. ΔT^{lo}. (D-I) Absolute MFI values of the different antibody classes and IgG subclasses. Data represent the raw data used for the ratios shown in (A). Each symbol represents an individual donor (n=27 ΔT^{lo}, n=11 ΔT^{hi}). Data are presented as median ± IQR (C-I). Statistical comparisons were performed using Mann-Whitney U tests, ***p*<0.01, ****p*<0.001, *****p*<0.0001.

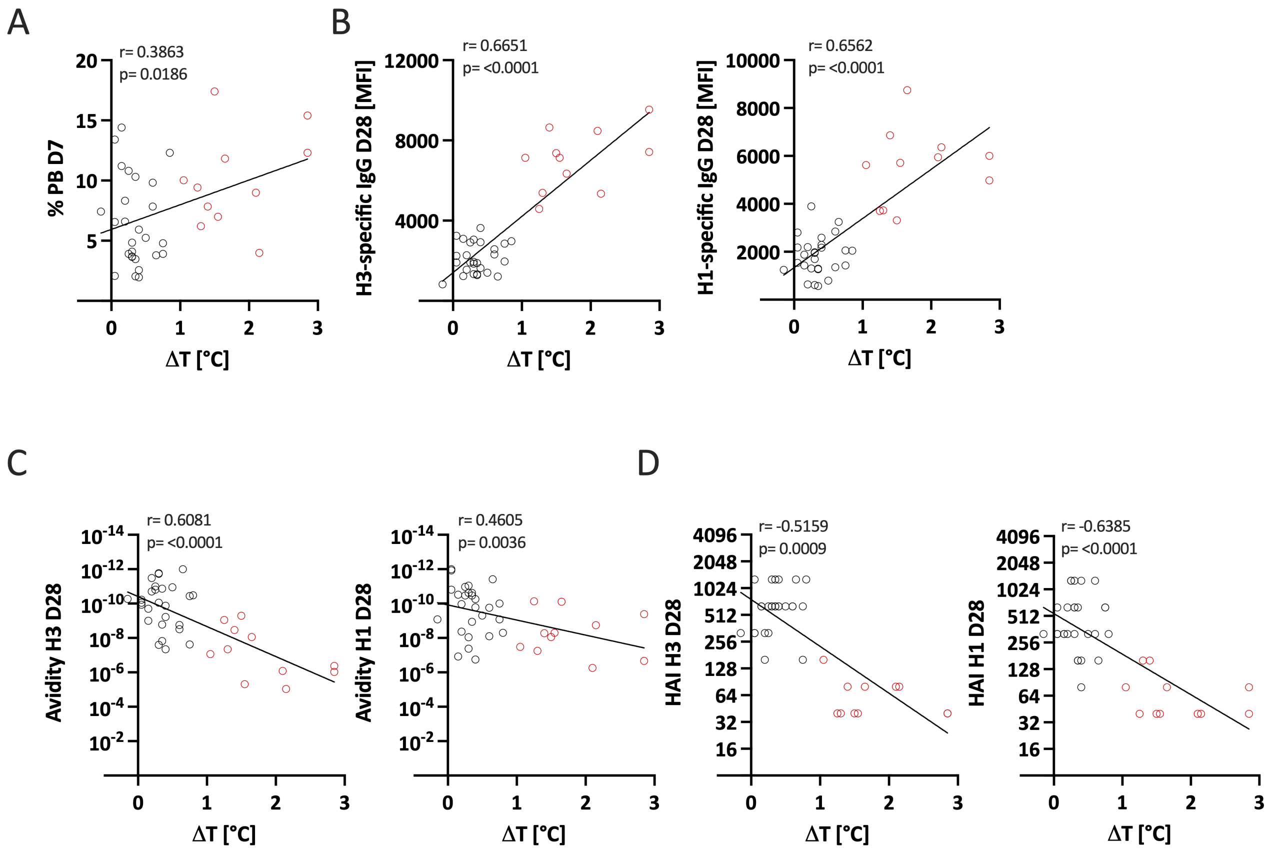

Supplementary Figure 14: Correlations of local thermal reactions and adaptive vaccine response. Correlation analyses were performed using simple linear regression; r and p values were calculated using Pearson's correlation (n = 38). ΔT was correlated with (A) frequency of plasmablasts in the peripheral blood on day 7 post vaccination; (B) HAI titers against H3 (left) and H1 (right) on day 28 post-vaccination; (C) Antibody avidity against H3 (left) and H1 (right) on day 28 post-vaccination; and (D) H3- (left) and H1-specific IgG (right) on day 28 post-vaccination. PB= plasmablast, H= hemagglutinin, HAI= hemagglutinin inhibition

Supplementary Tables

Supplementary Table 1: Antibodies used in *in vitro* experiments

Antibodies	Fluorochromes	Clone	Source
In vitro<br>Monocyte phenotype			
anti-human CD3	Alexa Fluor 700	OKT3	Biolegend
anti-human CD19	Brilliant Violet 510	HIB19	Biolegend
anti-human CD14	Brilliant Ultraviolet 395	63D3	BD Horizon™
anti-human CD16	Brilliant Ultraviolet 496	3G8	BD Horizon™
anti-human HLA DR	PE	L243	Biolegend
anti-human CD86	PeCy7	FUN-1	Biolegend
anti-human CD69	FITC	FN50	Biolegend
In vitro<br>B cell phenotype			
anti-human CD3	FITC	UCHT1	Biolegend
anti-human CD19	Alexa Fluor 700	HIB19	Biolegend
anti-human CD20	Brilliant Violet 510	2H7	Biolegend
anti-human CD27	PE	M-T271	Biolegend
anti-human CD38	APC	HIT2	Biolegend
anti-human IgD	PeCy7	IA6-2	Biolegend
anti-human CD69	Brilliant Violet 421	FN50	Biolegend

Supplementary Table 2: Antibodies used for *in vivo* phenotyping

Antibodies	Fluorochromes	Clone	Source
In vivo<br>Monocyte phenotype			
anti-human CD3	Alexa Fluor 700	OKT3	Biolegend
anti-human CD19	Brilliant Violet 510	HIB19	Biolegend
anti-human CD14	FITC	63D3	Biolegend
anti-human CD16	Brilliant Ultraviolet 496	3G8	BD Horizon™
anti-human HLA DR	PE	L243	Biolegend
anti-human CD86	PeCy7	FUN-1	Biolegend
In vivo<br>B cell phenotype			
anti-human CD3	FITC	UCHT1	Biolegend
anti-human CD19	Alexa Fluor 700	HIB19	Biolegend
anti-human CD20	Brilliant Violet 510	2H7	Biolegend
anti-human CD27	PE	M-T271	Biolegend
anti-human CD38	APC	HIT2	Biolegend
anti-human IgD	PeCy7	IA6-2	Biolegend
In vivo<br>T cell phenotype			
anti-human CD3	Alexa Fluor 700	OKT3	Biolegend
anti-human CD4	Brilliant Violet 510	SK3	Biolegend
anti-human CD8	PeCy7	SK1	Biolegend
anti-human CD45RO	eFluor 450	UCHL1	Invitrogen™
anti-human CD27	APC	M-T271	Biolegend
anti-human CD25	Brilliant Violet 605	BC96	Biolegend
anti-human CD95	PE	DX2	Biolegend
anti-human CD127	Brilliant Violet 711	A019D5	Biolegend
anti-human CXCR5	Alexa Fluor 488	J25D4	Biolegend
